# The Effect of Molecular Structure and Environment on the Miscibility and Diffusivity in Polythiophene-Methanofullerene Bulk Heterojunctions: Theory and Modeling with the RISM Approach

**DOI:** 10.3390/polym8040136

**Published:** 2016-04-09

**Authors:** Alexander E. Kobryn, Sergey Gusarov, Karthik Shankar

**Affiliations:** 1 National Institute for Nanotechnology, National Research Council Canada, 11421 Saskatchewan Drive, Edmonton, AB T6G 2M9, Canada; alex.kobryn@nrc-cnrc.gc.ca (A.E.K.); sergey.gusarov@nrc-cnrc.gc.ca (S.G.); 2 Department of Electrical and Computer Engineering, University of Alberta, Edmonton, AB T6G 2V4, Canada

**Keywords:** organic photovoltaics, structural and dynamical properties, PCBM, P3HT, P3BT, RISM

## Abstract

Although better means to model the properties of bulk heterojunction molecular blends are much needed in the field of organic optoelectronics, only a small subset of methods based on molecular dynamics- and Monte Carlo-based approaches have been hitherto employed to guide or replace empirical characterization and testing. Here, we present the first use of the integral equation theory of molecular liquids in modelling the structural properties of blends of phenyl-C${}_{61}$-butyric acid methyl ester (PCBM) with poly(3-hexylthiophene) (P3HT) and a carboxylated poly(3-butylthiophene) (P3BT), respectively. For this, we use the Reference Interaction Site Model (RISM) with the Universal Force Field (UFF) to compute the microscopic structure of blends and obtain insight into the miscibility of its components. Input parameters for RISM, such as optimized molecular geometries and charge distribution of interaction sites, are derived by the Density Functional Theory (DFT) methods. We also run Molecular Dynamics (MD) simulation to compare the diffusivity of the PCBM in binary blends with P3HT and P3BT, respectively. A remarkably good agreement with available experimental data and results of alternative modelling/simulation is observed for PCBM in the P3HT system. We interpret this as a step in the validation of the use of our approach for organic photovoltaics and support of its results for new systems that do not have reference data for comparison or calibration. In particular, for the less-studied P3BT, our results show that expectations about its performance in binary blends with PCBM may be overestimated, as it does not demonstrate the required level of miscibility and short-range structural organization. In addition, the simulated mobility of PCBM in P3BT is somewhat higher than what is expected for polymer blends and falls into a range typical for fluids. The significance of our predictive multi-scale modelling lies in the insights it offers into nanoscale morphology and charge transport behaviour in multi-component organic semiconductor blends.

## 1. Introduction

Organic Photovoltaics (OPVs) are considered as a promising next generation alternative power source that relies on sunlight due to their light weight, thin size, compatibility with flexible substrates and relatively simple fabrication advantages. In addition, OPVs demonstrate favourable electronic properties and component versatility. Because of the promise to be solution processable and applicable for a wide variety of flexible devices, their industrial-scale production and installation costs are expected to be low. Finally, steady improvements in OPVs’ efficiency have attracted great attention in recent years [1,2,3,4,5].

Thin film morphology is a key factor determining the performance of bulk heterojunction organic solar cells through its influence on charge separation, charge transport and recombination losses in donor-acceptor blends [6,7,8,9,10,11,12,13,14,15]. Even when conjugated polymers and/or small molecules with good charge carrier mobilities and the right energy levels for light absorption and device operation are chosen and used in binary blends, the efficiency of fabricated devices is poor (<0.5%) in the absence of a suitable morphology with percolating nanoscale domains. Unfortunately, whether or not a desirable morphology forms has been primarily ascertainable through empirical trial and error. In response, there have been attempts to use rod-coil diblock copolymers to deterministically engineer the nanoscale morphology of the active layer, with mixed results [16,17,18].

**Figure 1:**
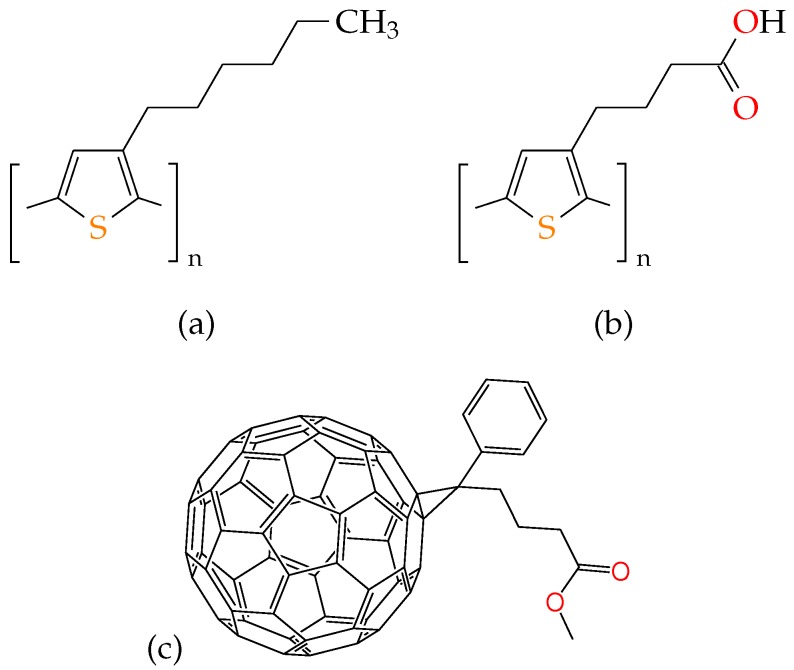
Schematic chemical structures of polymeric materials P3HT (**a**), P3BT (**b**) and PCBM (**c**). For better contrast, sulphur sites are marked orange, and oxygen sites are marked red.

Carboxylated poly(3-hexylthiophene-2,5-diyl) derivatives (P3HT) (Figure 1a) are interesting for a number of reasons. Not only have they been shown to effectively sensitize TiO${}_{2}$ in dye-sensitized solar cells, they have also been found to form blends with phenyl-C${}_{61}$-butyric acid methyl ester (PCBM) (Figure 1c) with efficiencies as high as 3.7% [19,20,21]. Unlike P3HT, whose solubility is highest in non-polar or weakly-polar solvents, its carboxylated polythiophene derivative poly(3-carboxybutylthiophene) (P3BT-COOH or P3BT for short) (Figure 1b) has the highest solubility in polar aprotic solvents, and the morphology resulting from such solution-processed thin films is not well-studied. In one work, well-defined rod-like structures have been reported in carboxylated polythiophenes and blend films thereof due to their enhanced self-aggregation tendencies [21].

Molecular dynamics simulations have been used in prior reports to identify the effect of side-chains on the crystallinity, thermal conductivity and structure-related fluorescence in conjugated polymers [22,23]. Statistical mechanics-based methods have successfully been used to identify *π*–*π*-stacking interactions between conjugated polymer chains through features in Radial Distribution Functions (RDFs) and to even quantify crystalline domain sizes occurring as a result [24]. However, very little work has been reported on exploring the effects of other types of interactions, such as hydrogen bonds, halogen bonds and dipole-dipole interactions between different chains in the same polymer and between the polymer and the small molecule occurring due to substituents and functional groups. Such interactions can also strongly influence blend miscibility, blend morphology and crystallite sizes.

In this context, we use the integral equation theory of molecular liquids in the interaction site formalism, also known as the reference interaction site model (RISM) [25,26], to elucidate RDFs in single-component films and blends with PCBM of carboxylated polythiophene derivative P3BT and compare them to the same in P3HT neat films and P3HT-PCBM blend films. RISM is a statistical mechanical theory to describe the equilibrium structure of molecular liquids in terms of the site-site pair correlation functions, from which all of the thermodynamic quantities can be derived. It provides a detailed microscopic insight into the organization of solvent molecules in the solvation shell structure and their contribution to the solvation thermodynamics. Being free from limitations inherent in heuristic theories used in standard simulation techniques, RISM and its generalizations have been applied successfully to almost the entire spectrum of chemical and physical processes taking place in a solution from chemical reactions to the molecular recognition by protein [26].

This article is organized as follows. For reader convenience, a brief summary of RISM theories is provided in Section 2. In Section 3, we summarize the modelling and simulation details and emphasize the role played by Density Functional Theory (DFT) to determine the optimized geometry and charge distribution of the studied molecules. An explanation for the choice of the force field and other input parameters is also provided. The main results are found in Section 4. Here, we present and analyse the solutions to 1D- and 3D-RISM equations in terms of 1D- and 3D-RDFs, use this analysis to discuss the miscibility of the components and touch on the dynamics in terms of the diffusion coefficients that we calculate from MD simulations. Whenever possible, we compare our results with to data of experimental measurements and/or numerical simulations available from the literature. The summary of our study is put in Section 5. A flowchart diagram showing the relation between DFT, RISM and MD in a multiscale description of microscopic structural and dynamical properties of studied systems, interaction site labelling and force field data, as well as a number of 1D-RDFs for selected interaction sites are provided in the Supplementary Materials (SM).

## 2. A Brief Outline of RISM

Static structural and thermodynamic properties of fluids can be conveniently described in terms of density fluctuations. This can be done, in particular, with the use of the Ornstein–Zernike (OZ) equation [26]. For uniform and isotropic fluids, it can be formulated from the grand partition function by functional differentiation in the spirit of the density functional theory and reads:

(1) $$h\left(r\right)=c\left(r\right)+\rho \int dr^{\prime }c(|r-r^{\prime }\left|\right)h\left(r^{\prime }\right),$$

where h(r)=g(r)−1 is a total correlation function, g(r) is a binary (or pair) correlation function, c(r) is a direct correlation function, *r* is the distance between molecules and ρ is the fluid density. The convolution integral in the r.h.s of the OZ equation represents the indirect correlation contribution to the total correlation function. Typically, the OZ equation is solved by taking the Fourier transform of both sides of the equation and obtaining the algebraic relation between the Fourier transforms $\tilde{h}\left(k\right)$ and $\tilde{c}\left(k\right)$, where *k* is a wave number in the reciprocal space. Since both h(r) and c(r) are unknown, in order to be solved, the OZ equation should be complemented by another equation, which relates h(r) and c(r). In general form, this equation, the closure, reads:

(2) c(r)=exp{−βϕ(r)+t(r)+b(r)}−1−t(r),

where $\beta =1/k_{B}T$ ($k_{B}$ is the Boltzmann constant and *T* is thermodynamic temperature), ϕ(r) is interaction potential, t(r)=h(r)−c(r), and b(r) is a functional of h(r) called a bridge function. The exact structure of b(r) can be obtained for special cases only; in all other cases, the OZ Equation (1) is solved with taking the closure Equation (2) with a certain approximation for b(r). The often used approximations are the hypernetted-chain (HNC) approximation, when b(r) is neglected, the Percus–Yevick (PY) approximation, with the further linearization of exp{t(r)}, and mean spherical approximation (MSA), when the closure is built on the use of the attractive (or repulsive) tail of the potential of interaction. The approximation proposed by Kovalenko and Hirata (KH) hybridizes the HNC with the MSA closures and reads:

(3) $$h\left(r\right)=\left\{\begin{array}{ccc} \exp\{-\beta \phi (r)+t(r)\}-1 & \mathrm{for} & h(r)⩽0,\\ -\beta \phi (r)+t(r) & \mathrm{for} & h(r)>0. \end{array}\right.$$

This closure utilizes the advantages and discards the disadvantages inherent in HNC and MSA closures. In the integral equation theory of liquids, now, it has become a standard.

In anisotropic homogeneous liquids consisting of polyatomic molecules interaction between a pair of molecules depend not only on the centre to centre distance *r*, but also on their mutual orientations in space $\Omega_{1}$ and $\Omega_{2}$. Taking this dependence into account brings one to the generalization of the OZ equation in the form [26,27,28]:

(4) $$h\left(12\right)=c\left(12\right)+\frac{\rho }{\Omega }\int d3\;c\left(13\right)h\left(32\right),$$

in which Ω is the normalization constant for the angular coordinates and function arguments denote the totality of both linear and angular coordinates describing positions and mutual orientations of molecules. The integration is carried over the entire space and the orientation of the third molecule. For inhomogeneous systems, such as gas-liquid or liquid-solid interfacial regions, the dependence of functions on the relative distance between molecule centres is replaced by the dependence on individual centre positions. The solution to the orientation-dependent OZ equation can be approached, in particular, through the spherical harmonic expansion of the correlation functions [28].

For molecules with complex geometries, it is sometimes useful to introduce correlation functions for their interaction sites. These sites may be the nuclei themselves or sites at arbitrary locations within the molecule (e.g., sites associated with CH${}_{2}$ or CH${}_{3}$ groups in the united atom models of hydrocarbons). Interaction sites within the molecule are labelled by Greek letters and, so, the correlation functions. The statistical interpretation of the site-site pair correlation function $g^{\alpha \gamma }\left(r_{\alpha \gamma }\right)$ is straightforward and defines it as being proportional to the probability density of finding the γ-site of some molecule at a distance $r_{\alpha \gamma }$ from the α-site of some different molecule. By fixing the distance between the interaction sites (rigid molecules) and averaging the functions over orientations, the equation for correlation functions is obtained in the form that is known as the Reference Interaction Site Model (RISM, or 1D-RISM) [26]:

(5) h=ω*c*ω+ρω*c*h,

where boldface letters denote matrices consisting of the site-site correlation functions $h^{\alpha \gamma }\left(r_{\alpha \gamma }\right)$ and $c^{\alpha \gamma }\left(r_{\alpha \gamma }\right)$, ρ is a diagonal matrix with the density, * denotes the convolution integral and the matrix product and ω is written as:

(6) $$\omega^{\alpha \gamma }\left(r_{\alpha \gamma }\right)=\delta_{\alpha \gamma }\delta \left(r_{\alpha \gamma }\right)+(1-\delta_{\alpha \gamma })s^{\alpha \gamma }\left(r_{\alpha \gamma }\right).$$

Here, $\delta_{\alpha \gamma }$ is the Kronecker delta; δ(r) is the Dirac delta function; and $s^{\alpha \gamma }\left(r_{\alpha \gamma }\right)$ is the intramolecular correlation functions between sites α and γ. For a rigid molecule, it is written as:

(7) $$s^{\alpha \gamma }\left(r_{\alpha \gamma }\right)=\frac{1}{4\pi \ell_{\alpha \gamma }^{2}}\delta \left(r-\ell_{\alpha \gamma }\right),$$

where $\ell_{\alpha \gamma }$ is the bond length or the distance constraint of the site pair α and γ. The RISM Equation (5) can be solved, in particular, with the use of the Fourier transform. In order to be solved, it has to be complemented with the closure relation in the interaction site representation. In the case of a multicomponent mixture, all of the matrices obtain two more indices to label species: e.g., $h_{ab}^{\alpha \gamma }\left(r\right)$ means that index *a* labels the α-th site in a molecular species *a*, *etc*. This also makes the diagonal matrix ρ consisting of the density of each molecular species, $\rho_{ab}^{\alpha \gamma }=\delta_{\alpha \gamma }\delta_{ab}\rho_{a}$. The RISM Equation (5) was originally obtained for generic liquids of polyatomic molecules. A number of generalizations for specific systems was suggested later. Some well-known versions include, in particular, DRISM, a dielectrically consistent version of RISM for polar liquids [29,30], and PRISM, a polymer RISM for liquids of polymers [31,32,33,34]. The former theory is analytically renormalized to ensure proper electrostatic asymptotics of site-site RDFs in electrolyte solution at a finite concentration and is capable of treating various liquids, mixtures and solutions comprising nonpolar, polar and ionic molecular species of a given composition in a wide range of thermodynamic conditions and the local environment. The latter is a model of a homopolymer melt or polymer mixture consisting of completely flexible chains described by a Gaussian intramolecular correlation function with the bond length between the nearest neighbour beads taken to be equal to the size of polymer repeat units. In a somewhat more realistic PRISM approach, the intramolecular correlation function is written for the freely-joined chain model in which the chains are made up of rigid bonds connected by freely-rotating joints.

In the 1D-RISM theories outlined above, the site-site correlation functions are averaged over the orientations of molecules. In order to have a more detailed picture of the solvation shell around the solute, the solvation structure should be represented by the probability density of finding site α of solvent molecules at 3D space position r around the solute molecule, $\rho^{\alpha }g^{\alpha }\left(r\right)$, which is determined by the average number density $\rho^{\alpha }$ in the solution bulk times the 3D distribution function $g^{\alpha }\left(r\right)$ of solvent site α. The latter indicates site density enhancement when $g^{\alpha }\left(r\right)>1$ or depletion when $g^{\alpha }\left(r\right)<1$ relative to the average density at a distance from the solute in the solution bulk, where $g^{\alpha }\to 1$. The 3D distribution functions of solvent interaction sites are obtained from the 3D-RISM integral equation [35]:

(8) $$h^{\alpha }\left(r\right)=\sum_{\gamma }\int dr^{\prime }c^{\gamma }\left(r-r^{\prime }\right)\chi^{\gamma \alpha }\left(r^{\prime }\right),$$

where indices α and γ enumerate all sites on all sorts of solvent species, $h^{\alpha }\left(r\right)$ is the 3D total correlation function of solvent site α related to the 3D site distribution function by $h^{\alpha }\left(r\right)=g^{\alpha }\left(r\right)-1$, $c^{\alpha }\left(r\right)$ is the 3D direct correlation function having the asymptotics of the solute-solvent site interaction potential, $c^{\alpha }\left(r\right)\to -\beta \phi^{\alpha }\left(r\right)$, and $\chi^{\alpha \gamma }\left(r\right)=\omega^{\alpha \beta }\left(r\right)+\rho^{\alpha }h^{\alpha \beta }\left(r\right)$ is the site-site susceptibility of pure solvent and is an input from 1D-RISM theory. To be solved, Equation (8) has to be complemented by the closure relation. We use the 3D-KH closure approximation [35]:

(9a) $$\begin{array}{ccc} g^{\alpha }\left(r\right) & = & \left\{\begin{array}{ccc} \exp\{d^{\alpha }\left(r\right)\} & \mathrm{for} & d^{\alpha }\left(r\right)⩽0,\\ 1+d^{\alpha }\left(r\right) & \mathrm{for} & d^{\alpha }\left(r\right)>0, \end{array}\right. \end{array}$$

(9b) $$\begin{array}{ccc} d^{\alpha }\left(r\right) & = & -\beta \phi^{\alpha }\left(r\right)+h^{\alpha }\left(r\right)-c^{\alpha }\left(r\right), \end{array}$$

proven to be appropriate to describe various association effects in complex liquids and electrolyte solutions and in supramolecular synthetic, organic and biomolecular systems in solution. Equations (8) and (9) are referred to as the 3D-RISM-KH theory. Typically, they are solved numerically with the use of the 3D Fourier transform.

## 3. Modelling and Simulation Details

**Figure 2:**
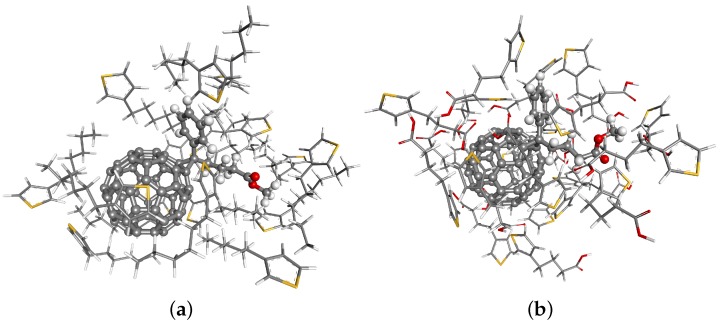
A visualization of DFT-optimized structures of clusters: (**a**) PCBM molecule surrounded by 16 P3HT monomers (520 interaction sites in total); (**b**) PCBM molecule surrounded by 10 P3BT monomers (508 interaction sites in total). Optimization has been performed with the use of the PBC functional [42] with OBS vdW correction [43] and the DND basis set.

We begin with geometry optimization of P3HT and P3BT monomers and polymers and PCBM molecules that have been carried out by DFT with the gradient-corrected functional of Hamprecht, Cohen, Tozer and Handy (HCTH) [36] and numerically-derived basis set DNP of double-zeta quality with polarization functions [37]. Core electrons were described by the density functional semi-core pseudopotentials [38]. The environment effects were taken into account by the Conductor-like Screening Model (COSMO) [39]. The charges were obtained by fitting them to reproduce the molecular Electrostatic Potential (ESP) [40] and used as input parameters in RISM. The computations were performed by using the DMol3 software from Accelrys Materials Studio [41] and graphical representations generated with the Materials Studio Visualizer. A view of final optimized geometries for P3HT and P3BT monomers and the PCBM molecule, as well as interaction sites’ ID numbers can be found in the SM. In addition, we have performed the DFT optimization of molecular clusters; see Figure 2. In these clusters, we were interested in checking the close contacts between interaction sites belonging to different species. For a distance criterion less than or equal to 0.8 relative to the sum of the van der Waals (vdW) radii of the interaction sites, the numbers of close distances are 23 and 16 for the PCBM-P3HT and PCBM-P3BT systems, respectively. Although the counts are different, we observe that the number of close contacts around the C${}_{60}$ part of the PCBM molecule is nearly the same in both cases: five and six, respectively. In such a way, the remaining close contacts are formed mostly around the tail of the PCBM molecule and for studied systems are largely different in numbers: 18 and 10, respectively. Such a big difference should strongly affect the behaviour of the systems on a larger scale. As we shall see later, the PCBM-P3HT system has variable miscibility, while P3BT seems to be a poor solvent for PCBM at all concentrations.

Parameters of the Lennard–Jones potential for interaction sites in the all-atom models of P3HT, P3BT and PCBM for RISM calculations were taken from the UFF [44,45,46]. Their actual numerical values together with interaction site Coulomb charges and Cartesian coordinates can be found in the SM. The calculations were performed for pure neat films of P3HT and P3BT monomers, dimers and trimers and blends of PCBM in P3HT and PCBM in P3BT monomers at various weight fractions (w.f.), starting from infinite dilution and up to 15% of PCBM, and at temperatures of 400, 450, 500, 550 and 600 K. Densities were adopted from two different sources: 0.933 [47] and 1.15 g/cm${}^{3}$ [48], for both P3HT and P3BT, and 1.3 g/cm${}^{3}$ for PCBM. In RISM equations, the dielectric properties of components are not considered; therefore, different dielectric constants of blend components do not affect the result. In the numerical aspect, the RISM equations were discretized on a uniform radial grid of 4096 nodes with 0.05-Å resolution and converged to a relative root-mean-square accuracy of $10^{-6}$ by using the Modified Direct Inversion in Iterated Space (MDIIS) accelerated numerical solver [35]. Similar set-ups for RISM or its modifications were successfully applied in our previous studies of both liquid and polymeric systems to describe their structure, explain the behaviour or to predict concentration- and thermodynamic-dependent properties [49,50,51,52,53,54,55,56,57].

## 4. Results and Discussions

Physically meaningful solutions to RISM equations were obtained at all temperatures for infinite dilution, 5%, 10% and 15% w.f. of PCBM in P3HT monomers and P3HT density 1.15 g/cm${}^{3}$. For higher concentrations, taken with the same increment rate of 5% and the same P3HT density or for any finite concentration and P3HT density 0.933 g/cm${}^{3}$, the determinant formed of the direct and intramolecular correlation functions in the reciprocal space [58] runs into negative values, which causes the expressions for free energy and pressure to diverge, although their analytical continuation can be set up. Solutions to RISM equations for these concentrations cannot be used for analysis of the system structure, as they exist in the form of slowly-decaying oscillations, which corresponds to long-range fluctuations of unstable homogeneous fluid with negative compressibility. This result is in very good agreement with the experimental study reported previously [59] in which a 19% *v/v* equilibrium concentration of PCBM in P3HT at 140 °C is interpreted as a point on the binodal curve of the composition-temperature phase diagram [60]. The authors hypothesize that experimentally-checked PCBM concentrations of 29.4% and 35.7% *v/v*, in which samples no crystals were observed, are metastable and that the concentration of 45.5% *v/v* is deeper in the two-phase region of the phase diagram, possibly close to the spinodal line that delineates the spontaneous unstable region in the phase diagram. In this respect, our simple theoretical model and the study [59] complement each other in the sense that the model captures the existence of an unstable region for some finite concentrations of PCBM in the P3HT blend that is observed experimentally. In earlier studies [61], the PCBM solubility limit, *i.e.*, the phase-separation point, was determined to be 30% w.f. The actual PCBM miscibility in amorphous portions of P3HT in a range between 10% and 20% of w.f. was reported in [62]. Later, it was found [63] that for a P3HT concentration greater that 42% w.f., the components are miscible. High miscibility and rapid interdiffusion for a blend of equal w.f. of PCBM and P3HT was experimentally observed and reported in [9]. This difference in numbers should be attributed to the fact that the fullerene-polymer system is not composed of two pure phases, but also contains an intermixed phases of amorphous and semicrystalline polymer and fullerene molecules [64]. The relative amount of semicrystalline and amorphous P3HT, *i.e.*, crystallinity, is determined between 40% and 60%, which decreases with increasing PCBM content, and PCBM is most probably embedded in the amorphous phase [64]. However, not all experiments can distinguish between the amorphous and the semicrystalline phases in P3HT. In the present theoretical model of the homogeneous mixture of PCBM in P3HT, the upper limit of the PCBM concentration happens to be around 20% w.f. This value is in agreement with the recent experimental report [65], where the maximal PCBM concentration in the amorphous P3HT interlayers is estimated between 20% and 52% w.f.

With this in mind, the PCBM in P3BT blend was studied within the same theoretical model. Except for infinite dilution, physically-meaningful solutions were not found either for the lower nor for the higher density of P3BT. This suggests that, as in the case of PCBM in P3HT, there is a region of unstable mixture, and it ranges for most of the finite concentrations of PCBM. Alternatively, one may think, e.g., about the impact of the force field used. For instance, in recent MD simulations [66], it was reported that amorphous blends of P3HT and C${}_{60}$ are either miscible or immiscible for typical blend compositions and processing conditions used in OPV, depending on which force fields are used. However, the lack of experimental data in the literature about PCBM in the P3BT system does not stimulate one to try different force fields, as the release of results prior to the measurements will only show the effect of the force fields and may not be conclusive about the actual system behaviour.

The solutions to RISM equations describing systems of P3HT or P3BT neat films and mixtures of PCBM in P3HT or P3BT were used to analyse the microscopic structure of these systems. Unlike the solid state, in which interaction sites are making only small oscillations around their equilibrium positions, interaction sites in liquids, solutions or melts are in diffusive motion of their molecules and change their positions continuously. For fullerene-based bulk-heterojunction films, it is well known that the fullerenes form clusters due to fullerene diffusion and agglomeration during thermal annealing. Owing to the thermal motion, the density of interaction sites varies as a function of space and time. The most basic concept in describing the liquid state is the local density, or the density field and its fluctuation. Site-site RDFs, g(r), are convenient instruments in this description, as they provide considerable insight into the liquid structure, at least on the level of pair correlations [26]. They tend to unity at large distances and vanish at small ones as a consequence of the strongly repulsive forces acting at small separations, while peaks and minima in g(r) represent “solvation shells” and density depletion, respectively, of neighbouring sites around the reference interaction site and set up a useful measure to describe the short to medium range structure of a liquid.

**Figure 3:**
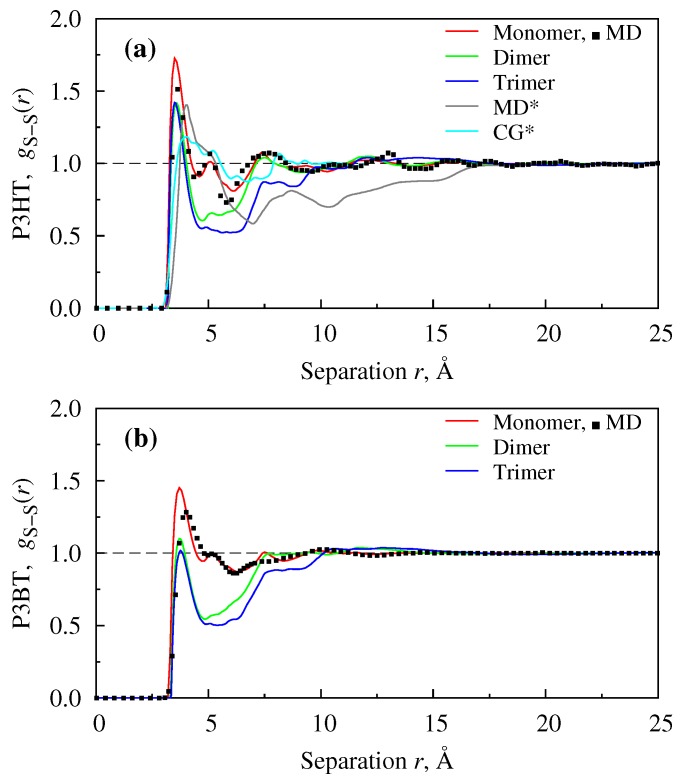
Radial Distribution Functions (RDFs) for S–S interaction sites belonging to neat films P3HT (**a**) and P3BT (**b**), modelled as monomers, dimers or trimers at 400 K. MD${}^{*}$ data are taken from [70] and CG${}^{*}$ from [24].

**Figure 4:**
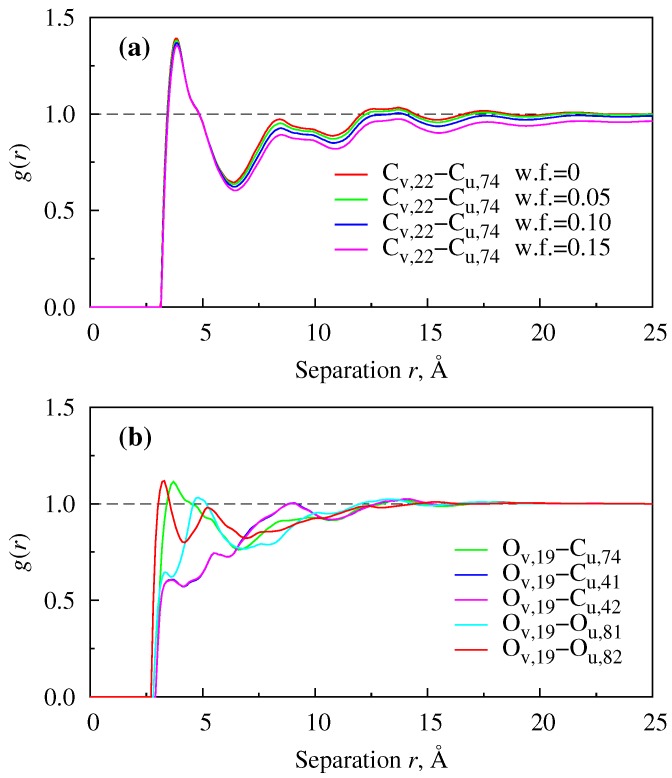
RDFs for X${}_{v}$–Y${}_{u}$ interaction sites where X${}_{v}$ belongs to P3HT (**a**) or P3BT (**b**) and Y${}_{u}$ belongs to PCBM, at 400 K. Numerical labels of interaction sites can be found in Supplementary Materials (SM).

Typical plots of site-site RDFs of the studied systems can be seen in Figure 3 and Figure 4. First, we compare the short-range structures for monomers (red line), dimers (green line) and trimers (blue line), as shown in Figure 3a,b. For monomers, the study is complemented with relatively short-run MD simulation (filled squares). Although the positions of the first peak approximately coincides for all “mers”, the separation with the next peak and the size of the separation well visibly increase with the polymerization degree. The latter is explained by the fact that in the case of dimers and trimers, the nearby space is occupied by the next repeat unit, which prevents other molecules from entering the area. This feature is common for both P3HT and P3BT neat films. For the P3HT material, we also include several results available from the literature. In particular, the grey line represents MD simulation data tracking the same interaction sites as in our study, and the bright blue line represents Dissipative Particle Dynamics (DPD) simulation data for a coarse-grained bead of a head-part of the molecule. Although the positions of peaks and their amplitudes may not coincide exactly in the former case, which can be explained, in particular, by the difference in the force field used, there is a visible correlation between them. In the latter case, the difference between RDFs is somewhat bigger, which is inevitable if the coarse-graining is done not by the structure matching procedure or refers to virtual interaction sites. At the same time, it is remarkable that the first peak position values obtained from the theory are close to the ones measured experimentally. In particular, the first peak position for P3HT is found at 3.75 Å when the system is studied at ρ=0.933 g/cm${}^{3}$ and at 3.5, 3.6, or 3.5 Å for monomers, dimers or trimers, respectively, studied at ρ=1.15 g/cm${}^{3}$. The experimentally-observed value is 3.8 Å [67] or falls into a range 3.81–3.87 Å [68], depending on the molecular weight of the system. For P3BT, the first peak position is found at 3.7 Å for monomers and dimers and 3.75 Å for trimers, all studied at ρ=1.15 g/cm${}^{3}$. The only experimental value found is 3.1 Å [67]; however, it was reported for a *non*-carboxylated P3BT. Here, it is worth noting that the dependence of the sensitivity in these kinds of measurements on a number of factors was touched on in a number of previous studies [67,68,69].

A representative plot of RDFs between interaction sites belonging to solvent and solute molecules (for which we use subscripts u and v, respectively) can be seen in Figure 4. In particular, RDFs for solute tail carbon (Site #22) – solvent carbon of the ring (Site #74) at various solute weight fractions are found in Figure 4a. The persistence of RDFs’ short- and medium-range patterns with solute concentration suggests that there can be a similar length scale strong mutual organization between solvent and solute molecules. Some representative RDFs in Figure 4b are for the system of PCBM in P3BT at infinite dilution and have a liquid-like structure, with a relatively weak first peak for most of the interaction site pairs. Finite concentration results were not found. This excludes possible short to medium range mutual structural organization in such a system and suggests that for finite concentrations, the system is thermodynamically unstable or does not exist. An extensive set of various types of RDFs for both mixtures and neat films is put into SM.

**Figure 5:**
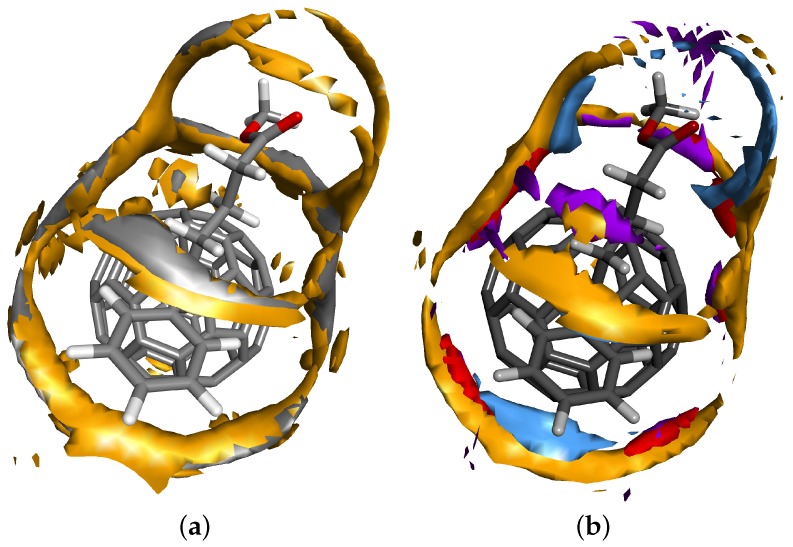
3D solvation structure around the PCBM molecule in P3HT (**a**) and P3BT (**b**), respectively, at T=400 K, obtained with the use of the 3D-RISM-KH theory. Different colour isosurfaces around the PCBM molecule correspond to the probability of finding different interaction sites belonging to P3HT or P3BT, respectively. Only high values of the first solvation shell are shown. We use the following colours: dark yellow, sulphur of P3HT or P3BT; grey, carbon at the end of the hydrocarbon tail of P3HT; red, oxygen in the OH group of P3BT; purple, oxygen of P3BT; blue, hydrogen in the OH group of P3BT. The 3D distribution of interaction sites demonstrates preferential mutual orientation of solute and solvent molecules in solution and defines the morphology of the system on the nanoscale.

RDFs obtained in the RISM integral equation theory of fluid are averaged over orientations in space. For the general analysis of the short-range fluid structure, this may be sufficient. However, for molecules with complex geometries, as in our case, 3D distributions obtained from the 3D-RISM theory of solvation can provide additional morphological information about the solvent around the solute, including the preferential mutual orientation of molecules in space. In this respect, we run 3D-RISM-KH calculations for the studied systems at T=400 K. Figure 5a,b exhibits the isosurfaces of the 3D distribution functions of selected interactions sites from P3HT and P3BT monomers, respectively, showing the regions with the highest probability of finding the sites around the PCBM molecule. The numerical values of isosurfaces are 3.6 for both sulphur and tail end carbon sites of the P3HT molecule, while for the P3BT molecule, these values are three for sulphur, 2.6 for both oxygen sites and 2.2 for hydrogen in the OH group. It is seen well that in both cases all of the clouds appear mostly in the area between the buckyball part and the butyric acid methyl ester functional group part to form a sort of a belt with a few bridges over the functional group, but not in the opposite direction. By decreasing the isosurface numerical value, one can visualize the formation of the 3D solvation shell of the selected interaction site around the solute molecule. For our two different solvents, this process results in the following. In the case of P3HT, both sulphur and carbon clouds first extend around the functional group and then around the buckyball part of PCBM, to quickly form a solvation shell around the whole molecule. In the case of P3BT, to achieve a similar effect with the cloud for sulphur, the isosurface value should be lowered to a proportionally smaller value. The isosurfaces for oxygen and hydrogen in the OH group prefer to extend around the functional group, while for the other oxygen, the isosurface develops almost exclusively around the neck between the buckyball and functional group parts. The probability of the formation of the solvation shell around the whole solute molecule is expected to be somewhat low. It is clear then that the oxygen and the OH group of P3BT avoid close contact with the hydrophobic fragment of the PCBM molecule. This factor explains the lack of solutions to 1D-RISM equations at finite concentrations and suggests that the miscibility of the components will be low. Another observation from both 1D- and 3D-RISM studies is that the OH group may not form stable hydrogen bonds with PCBM oxygen sites simply because the formation does not occur at such high temperatures.

**Figure 6:**
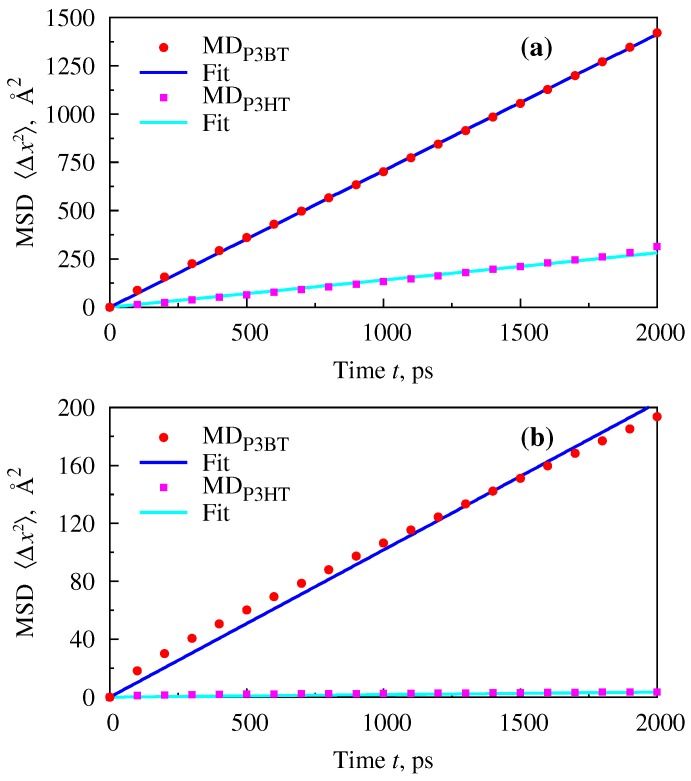
MD results for mean square displacement of PCBM in its blend with P3BT or P3HT monomers at infinite dilution and T=400 K. (**a**) $\rho_{\mathrm{blend}}=0.933$ g/cm${}^{3}$; (**b**) $\rho_{\mathrm{blend}}=1.15$ g/cm${}^{3}$. Symbols denote the Mean Square Displacement (MSD) values sampled from data every 200 time steps, and lines are least-squares Marquardt–Levenberg algorithm [71] linear fits to them.

Although the study by extensive simulation was not our primary intention, we did perform several MD runs, mostly with the purpose to check the solute dynamics at infinite dilutions and to compare it to the data from the literature. Dynamical properties cannot be acquired by the integral equation theory of molecular liquids and must be obtained either from a *dynamical* theory or simulation. In particular, we have been interested in Mean Square Displacement (MSD) of the interaction sites belonging to solute molecule, with its further use in the estimation of the translational diffusion coefficient. All of the systems were studied in NVT ensemble with UFF and the productive run t=4000 ps. To obtain the diffusion coefficient as accurately as possible, the statistical noise in the MSD *vs*. time plot should be minimized. In Materials Studios [41], to improve the statistics, the ensemble average is usually calculated using multiple time origins. If a simulation generates a trajectory containing *M* frames at time interval δt, the MSD is calculated by comparing each frame with the first frame to obtain the displacement of each site at any given time *t*. However, the quality of the statistics declines for longer times. For this reason, only the first half of the MSD *vs*. time plot is considered when determining the diffusion coefficient for small to medium-sized systems. In Figure 6, the MSDs of PCBM in its blend with P3HT or P3BT at T=400 K is plotted for the first 2000 ps and for two different densities (Subfigures (a) and (b), respectively).

One can see that linear fits approximate simulation data very well for the lower density and satisfactorily for the higher one. The linear behaviour of MSD *vs*. time justifies the quality of simulations and allows one to estimate the diffusion coefficient by following the standard phenomenological relation [72]. The results are summarized in Table 1. Some alternative studies available from the literature are included for the reader’s convenience and are used as the reference data. According to [73], diffusion coefficients in high molecular weight polymers are closer to those for liquids than to those for solids. The values of these coefficients vary strongly with concentration and temperature within the conventionally-defined range $10^{-9}-10^{-6}$ cm${}^{2}$/s. From Table 1, one can see that this is the case for the diffusion of PCBM in P3HT at both studied densities of the solvent and for diffusion of PCBM in P3BT at only the higher density of the solvent, while at the lower density, the diffusion is like the one in a liquid. This factor and previously-mentioned difficulty in finding physically meaningful solutions to RISM equations describing the finite concentration of PCBM in the P3BT blend suggest that the morphologies needed to produce good OPVs are far more demanding for PCBM-P3BT blends. One reason for this conclusion is the liquid-like behaviour of the solvent and elevated mobility of the solute, and a second reason is that good electron transport requires PCBM solubility in the semiconducting polymer matrix [61]. Below the miscibility threshold, hole-only conduction is expected in PCBM-P3BT blends containing a dominant proportion of P3BT, which leads to transport paths with dead-ends for photogenerated electrons. The final conclusion about that, however, should be based on the more rigorous study or measurements.

## 5. Summary

In summary, we have suggested a model to study the structural and dynamical properties of OPV materials based on the use of PCBM (acceptor) and P3HT or P3BT (donors). To describe the structural properties, our model refers to the integral equation theory of molecular liquids in terms of the interaction site representation, also known as RISM (or one of its generalizations [26]). As input parameters, it requires information about the geometric structure of molecules and potentials between interaction sites. The former is carried out within the frame of DFT. For the latter, the Coulomb part of the interaction potential is also obtained from DFT by fitting charges to reproduce the molecular electrostatic potentials, while the non-Coulomb part is adopted from UFF [44,45,46]. To our knowledge, this is the first report of using UFF force fields in RISM. It is also the first time of using RISM theory for OPV materials. On the RISM output, one obtains RDFs for all of the interaction sites and uses them to analyse the short-range structure as a function of composition and temperature. So far, we performed these simulations for neat films of P3HT and P3BT monomers, dimers and trimers and for blends of PCBM and P3HT or P3BT monomers. In the literature, the density of P3HT material varies; therefore, we did all of the modelling for different densities found in different sources [47,48]. The analysis of RISM results reveals the following. With UFF, the RISM model of the studied OPV materials is capable of describing their short-range structure, so that it correlates with MD and DPD simulations from the literature. In addition, the RISM model is very sensitive to the blend composition, which happens to be in very good agreement with experimental observations. The inability to find physically-meaningful solutions to RISM equations at certain solute concentrations is interpreted as an indicator of the existence of an unstable region on the composition-temperature phase diagram. In the case of PCBM in P3BT, the physically-meaningful solutions to RISM equations were found for the infinite dilution only, which suggests a much stronger tendency toward phase segregation in PCBM-P3BT blends. This suggestion was confirmed once again in the MD simulations that we run (also with the UFF) to quickly estimate the diffusion coefficients of PCBM solute in P3HT or P3BT solvents at infinite dilution. Quantitatively, the diffusion coefficients of PCBM in P3HT fall into the range of the ones that are typical for high polymers, while the diffusion coefficients of PCBM in P3BT are closer to the ones that are found for a diffusion in fluids [73].

## Figures and Tables

**Table 1:** Diffusion coefficients of PCBM in its blend with P3HT or P3BT obtained in previous studies at various temperatures *T* and finite concentrations of PCBM $\phi_{\mathrm{PCBM}}$ and estimated in the present study from MD simulations at infinite dilution. Here, w.f. stands for weight fraction and *v/v* indicates the volume fraction of a component per total volume. $\phi_{\mathrm{PCBM}}=0$ means infinite dilution.

System	*T*, K	$\phi_{\mathrm{PCBM}}$	*D*, cm${}^{2}$/s	Ref.
PCBM in P3HT	423	40% w.f	$2.5\times 10^{-10}$	[74]
	413	19% *v/v*	$2.5\times 10^{-10}$	[59]
	383	0.001% *v/v*	$1.5\times 10^{-9}$	[75]
	383	0.01% *v/v*	$1.0\times 10^{-9}$	[75]
	423	0.01% *v/v*	$5.0\times 10^{-9}$	[75]
	383	0.05% *v/v*	$5.3\times 10^{-10}$	[75]
	400	0 *a*	$2.6\times 10^{-6}$	MD
	400	0 *b*	$3.0\times 10^{-8}$	MD
PCBM in P3BT	400	0 *a*	$1.2\times 10^{-5}$	MD
	400	0 *b*	$1.6\times 10^{-6}$	MD

*a* $\rho_{\mathrm{blend}}=\rho_{\mathrm{solvent}}=0.933$ g/cm${}^{3}$, [47]; *b* $\rho_{\mathrm{blend}}=\rho_{\mathrm{solvent}}=1.150$ g/cm${}^{3}$, [48].

## Supplementary Materials

Supplementary Materials can be found at www.mdpi.com/2073-4360/8/4/136/s1.
