# Supplementary material for: The Effect of Molecular Structure and Environment on the Miscibility and Diffusivity in Polythiophene-Methanofullerene Bulk Heterojunctions: Theory and Modeling with the RISM Approach
Source: Polymers (Basel). 2016 Apr 9;8(4):136. doi: 10.3390/polym8040136 (PMC6432195; doi:10.3390/polym8040136)
Supplement: Supplementary file 1 [file polymers-08-00136-s001.pdf]

Supplementary Materials: The Effect of Molecular Structure and Environment on the Miscibility and Diffusivity in Polythiophene-Methanofullerene Bulk Heterojunctions: Theory and Modeling with the RISM Approach

Alexander E. Kobryn [1], Sergey Gusarov [1] and Karthik Shankar [1,2,*]

The diagrammatic summary of the suggested multiscale modeling approach and its steps is presented in Figure S1.

All the details of the UFF force fields [1–3] used for P3HT, P3BT, and PCBM molecules can be found in Tables S1 and S2, respectively, while numerical labels of all the interaction sites can be identified from Figure S2.

Plots of 1D-RDFs of selected interaction sites are displayed in Figures S3–S21.

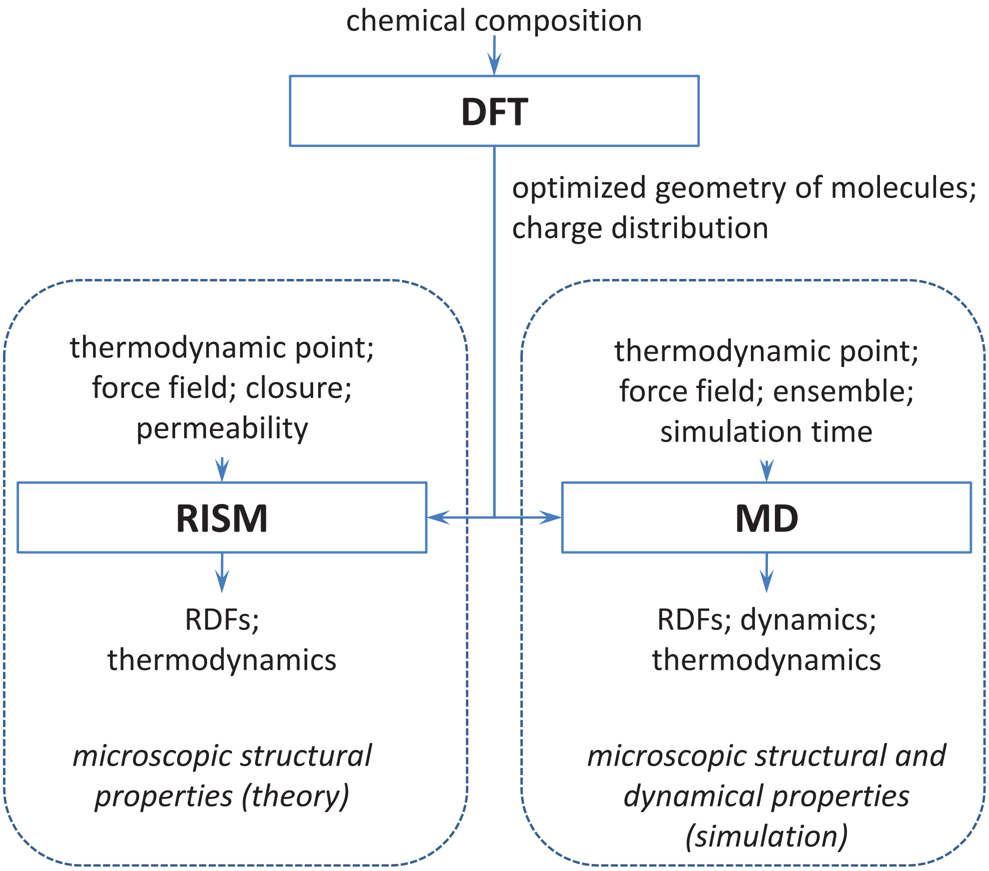

Figure S1. Flowchart diagram of multiscale modeling steps with indication of what is required for input and what is obtained in the output. The principal modeling steps are marked in bold font and include: DFT—density functional theory of atoms and molecules; RISM—reference interaction site model in the interaction site representation; MD—molecular dynamics simulation. Results from RISM and MD can be compared with each other for validation, or can be used as the input in next steps of hierarchical multiscale modeling, e.g., coarse-grained modeling, as it was demonstrated in the previous study [4].

Table S1. Parameters of the site-site interaction potential and Cartesian coordinates of interaction sites for solvents P3HT and P3BT; a.u. stands for "atomic units".

Site (#)	z_α (a.u.)	σ_α (Å)	ϵ_α (kcal/mol)	x (Å)	y (Å)	z (Å)	Site (#)	z_α (a.u.)	σ_α (Å)	ϵ_α (kcal/mol)	x (Å)	y (Å)	z (Å)
			**P3HT**							**P3BT**			
H(1)	0.190	2.571	0.044	−5.323210	2.013530	−0.001272	H(1)	0.193	2.571	0.044	−5.267730	2.153320	−0.017008
C(2)	−0.483	3.431	0.105	−4.268130	1.770450	−0.000657	C(2)	−0.483	3.431	0.105	−4.218730	1.885390	−0.005718
S(3)	0.444	3.595	0.274	−3.757420	0.041335	0.003568	S(3)	0.456	3.595	0.274	−3.747160	0.147005	0.051018
C(4)	−0.529	3.431	0.105	−2.033350	0.575168	0.002399	C(4)	−0.532	3.431	0.105	−2.012380	0.639616	0.042528
C(5)	0.129	3.431	0.105	−1.908470	1.941040	−0.000563	C(5)	0.129	3.431	0.105	−1.857800	2.002030	0.001552
C(6)	−0.100	3.431	0.105	−3.190930	2.610810	−0.002157	C(6)	−0.100	3.431	0.105	−3.123110	2.701710	−0.026101
H(7)	0.194	2.571	0.044	−1.254320	−0.177004	0.003992	H(7)	0.204	2.571	0.044	−1.252180	−0.131352	0.071711
H(8)	0.162	2.571	0.044	−3.288030	3.694650	−0.004900	H(8)	0.163	2.571	0.044	−3.196050	3.786710	−0.062337
C(9)	−0.381	3.431	0.105	−0.599443	2.702510	−0.002200	C(9)	−0.385	3.431	0.105	−0.531235	2.731720	−0.017419
C(10)	−0.335	3.431	0.105	0.670060	1.834920	−0.000135	C(10)	−0.335	3.431	0.105	0.703159	1.818000	0.044555
C(11)	−0.313	3.431	0.105	1.958580	2.678080	−0.001352	C(11)	−0.437	3.431	0.105	2.009750	2.624300	0.033897
C(12)	−0.312	3.431	0.105	3.242550	1.829410	0.000737	C(12)	0.388	3.431	0.105	3.248250	1.740200	0.075868
H(13)	0.180	2.571	0.044	−0.577513	3.367530	−0.883356	H(13)	0.182	2.571	0.044	−0.479339	3.351200	−0.930591
H(14)	0.180	2.571	0.044	−0.577718	3.371260	0.876112	H(14)	0.181	2.571	0.044	−0.505203	3.440350	0.829173
H(15)	0.169	2.571	0.044	0.666378	1.174620	−0.883396	H(15)	0.201	2.571	0.044	0.704326	1.122350	−0.806546
H(16)	0.169	2.571	0.044	0.665855	1.178030	0.885673	H(16)	0.200	2.571	0.044	0.670571	1.197390	0.951450
H(17)	0.159	2.571	0.044	1.960760	3.341940	0.880620	H(17)	0.205	2.571	0.044	2.037780	3.314100	0.895298
H(18)	0.159	2.571	0.044	1.961390	3.338590	−0.885844	H(18)	0.206	2.571	0.044	2.059990	3.255390	−0.870790
H(19)	0.157	2.571	0.044	3.243280	1.165490	−0.881301	O(19)	−0.477	3.118	0.060	4.476770	2.429610	0.083157
H(20)	0.157	2.571	0.044	3.242590	1.168650	0.885159	O(20)	−0.323	3.118	0.060	3.253060	0.508318	0.100775
C(21)	−0.320	3.431	0.105	4.529580	2.675470	−0.000331	H(21)	0.364	2.571	0.044	4.361770	3.414490	0.062320
C(22)	−0.474	3.431	0.105	5.808490	1.821800	0.001180							
H(23)	0.159	2.571	0.044	4.525500	3.336210	−0.883555							
H(24)	0.159	2.571	0.044	4.525200	3.338940	0.880836							
H(25)	0.159	2.571	0.044	5.848470	1.171870	−0.886399							
H(26)	0.159	2.571	0.044	5.848340	1.174690	0.890825							
H(27)	0.162	2.571	0.044	6.710770	2.450410	0.000240							

Table S2. Parameters of the site-site interaction potential and Cartesian coordinates of interaction sites for solute PCBM; a.u. stands for "atomic units".

Site (#)	z_α (a.u.)	σ_α (Å)	ϵ_α (kcal/mol)	x (Å)	y (Å)	z (Å)	Site (#)	z_α (a.u.)	σ_α (Å)	ϵ_α (kcal/mol)	x (Å)	y (Å)	z (Å)
			**Sites 1–44**							**Sites 45–88**			
C(1)	−0.003	3.431	0.105	0.239761	−0.163784	0.177104	C(45)	−0.005	3.431	0.105	−2.243130	1.365080	5.161820
C(2)	−0.002	3.431	0.105	1.693010	−0.256775	0.254974	C(46)	−0.002	3.431	0.105	−1.718740	2.667430	5.129510
C(3)	−0.002	3.431	0.105	2.231740	1.097590	0.246468	C(47)	−0.003	3.431	0.105	0.273596	3.929950	5.195550
C(4)	0.006	3.431	0.105	1.122140	2.031090	0.161378	C(48)	−0.004	3.431	0.105	1.673130	3.847870	5.293340
C(5)	0.001	3.431	0.105	−0.127606	1.242450	0.115378	C(49)	0.001	3.431	0.105	3.829750	0.982626	5.499140
C(6)	0.015	3.431	0.105	−1.290680	1.698010	0.735482	C(50)	−0.002	3.431	0.105	3.487320	2.341820	5.442880
C(7)	−0.006	3.431	0.105	−0.555260	−1.077810	0.885224	C(51)	−0.001	3.431	0.105	0.575536	−1.479810	6.168050
C(8)	−0.003	3.431	0.105	2.297150	−1.250180	1.036310	C(52)	−0.002	3.431	0.105	−1.624490	0.342919	5.998970
C(9)	−0.006	3.431	0.105	3.365090	1.405340	1.017310	C(53)	0.004	3.431	0.105	−0.557970	2.997280	5.947210
C(10)	0.018	3.431	0.105	1.178640	3.255800	0.822507	C(54)	−0.004	3.431	0.105	2.291440	2.824290	6.130230
C(11)	−0.002	3.431	0.105	3.464300	−0.925139	1.852420	C(55)	−0.002	3.431	0.105	2.993080	0.051765	6.248840
C(12)	−0.002	3.431	0.105	3.987080	0.375259	1.841450	C(56)	−0.003	3.431	0.105	0.034384	2.020310	6.758860
C(13)	−0.005	3.431	0.105	3.407050	2.655120	1.750340	C(57)	−0.001	3.431	0.105	1.489200	1.927470	6.848010
C(14)	0.004	3.431	0.105	2.321550	3.541470	1.668720	C(58)	−0.002	3.431	0.105	1.847450	0.516180	6.910170
C(15)	−0.082	3.431	0.105	−0.024283	3.941520	1.401490	C(59)	−0.001	3.431	0.105	0.613443	−0.265953	6.869460
C(16)	−0.072	3.431	0.105	−1.419610	3.058790	1.357590	C(60)	−0.002	3.431	0.105	−0.505412	0.663486	6.781080
C(17)	0.006	3.431	0.105	−2.073130	0.760527	1.520120	C(61)	−0.014	3.431	0.105	−1.297400	4.443550	0.695281
C(18)	−0.005	3.431	0.105	−1.737360	−0.602537	1.576890	C(62)	−0.286	3.431	0.105	−1.423130	4.640420	−0.839600
C(19)	−0.002	3.431	0.105	0.070116	−2.107360	1.709700	C(63)	−0.342	3.431	0.105	−0.459322	3.908300	−1.782690
C(20)	−0.003	3.431	0.105	1.467900	−2.191040	1.785930	H(64)	0.170	2.571	0.444	−1.328490	5.725470	−1.010130
C(21)	−0.003	3.431	0.105	−0.735954	−2.265210	2.917550	H(65)	0.162	2.571	0.444	−2.465410	4.382200	−1.096140
C(22)	−0.003	3.431	0.105	−1.855880	−1.334240	2.834520	C(66)	−0.324	3.431	0.105	−0.721488	4.235580	−3.270570
C(23)	−0.001	3.431	0.105	2.115360	−2.437320	3.067770	C(67)	0.405	3.431	0.105	0.220022	3.484870	−4.188110
C(24)	0.000	3.431	0.105	3.350230	−1.655770	3.108590	H(68)	0.240	2.571	0.444	−0.544357	2.825080	−1.668500
C(25)	−0.002	3.431	0.105	4.418130	0.998736	3.090670	H(69)	0.169	2.571	0.444	0.576768	4.179270	−1.538370
C(26)	−0.002	3.431	0.105	4.058550	2.411850	3.033970	C(70)	−0.129	3.431	0.105	−3.411800	5.525830	1.602130
C(27)	0.004	3.431	0.105	1.849310	4.223730	2.864980	H(71)	0.143	2.571	0.444	−3.956070	4.614470	1.356500
C(28)	0.023	3.431	0.105	0.410093	4.367720	2.772450	C(72)	−0.128	3.431	0.105	−4.094220	6.632380	2.124740
C(29)	0.023	3.431	0.105	−2.056030	2.806310	2.690360	H(73)	0.140	2.571	0.444	−5.170490	6.578400	2.282330
C(30)	0.003	3.431	0.105	−2.542070	1.441440	2.717830	C(74)	−0.132	3.431	0.105	−3.395170	7.803370	2.446610
C(31)	0.001	3.431	0.105	−0.112255	−2.499530	4.151610	H(75)	0.140	2.571	0.444	−3.926150	8.661850	2.854820
C(32)	−0.003	3.431	0.105	1.341870	−2.587240	4.227950	C(76)	−0.129	3.431	0.105	−2.010640	7.865200	2.242090
C(33)	−0.003	3.431	0.105	3.760040	−1.056360	4.308460	H(77)	0.141	2.571	0.444	−1.459610	8.771080	2.489500
C(34)	−0.002	3.431	0.105	4.303550	0.297635	4.299050	C(78)	−0.125	3.431	0.105	−1.329870	6.760420	1.716710
C(35)	−0.002	3.431	0.105	3.603820	3.068660	4.183440	H(79)	0.145	2.571	0.444	−0.253114	6.809980	1.556240
C(36)	−0.006	3.431	0.105	2.479600	3.995240	4.097830	C(80)	0.015	3.431	0.105	−2.023810	5.582070	1.393510
C(37)	0.011	3.431	0.105	−1.618950	3.399140	3.873190	O(81)	−0.435	3.118	0.060	0.347674	2.155150	−3.789320
C(38)	0.013	3.431	0.105	−0.368261	4.189320	3.914190	O(82)	−0.382	3.118	0.060	0.823888	3.949880	−5.170090
C(39)	−0.002	3.431	0.105	−2.310440	−0.678033	3.984280	C(83)	−0.194	3.431	0.105	1.287480	1.314670	−4.564140
C(40)	−0.005	3.431	0.105	−2.664510	0.736739	3.924650	H(84)	0.173	2.571	0.444	1.340660	0.379140	−4.003030
C(41)	0.000	3.431	0.105	1.767860	−1.959390	5.475050	H(85)	0.177	2.571	0.444	2.266020	1.804870	−4.620010
C(42)	0.000	3.431	0.105	2.950970	−1.208880	5.513960	H(86)	0.171	2.571	0.444	0.897337	1.153760	−5.575910
C(43)	−0.002	3.431	0.105	−1.665020	−0.922196	5.269810	H(87)	0.180	2.571	0.444	−0.601753	5.305910	−3.483660
C(44)	−0.002	3.431	0.105	−0.585919	−1.813870	5.351860	H(88)	0.183	2.571	0.444	−1.755580	3.956730	−3.534640

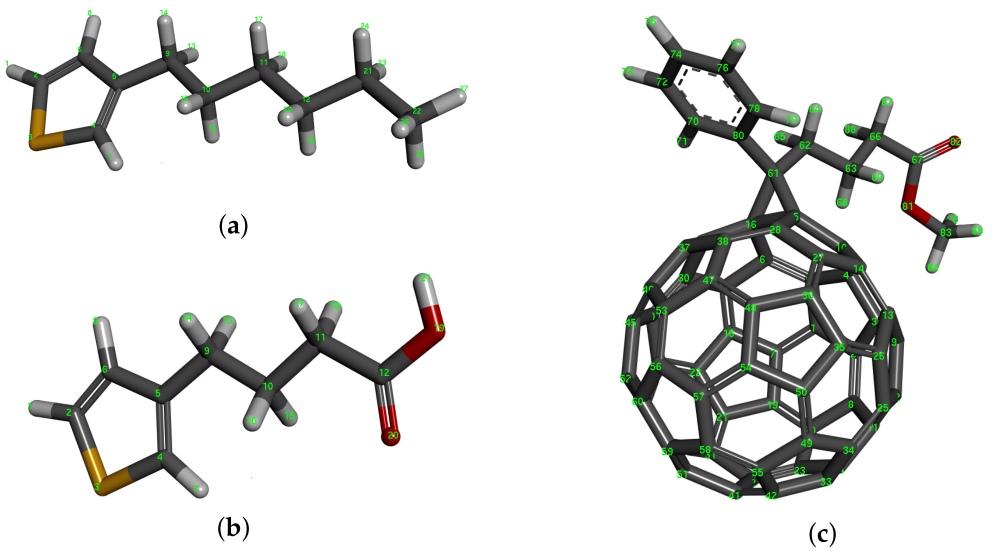

Figure S2. Schematic representation of P3HT, P3BT and PCBM molecules with site labels. Sulphur is marked yellow, carbon is dark gray, hydrogen is white, and oxygen is red. (**a**) P3HT; (**b**) P3BT; (**c**) PCBM.

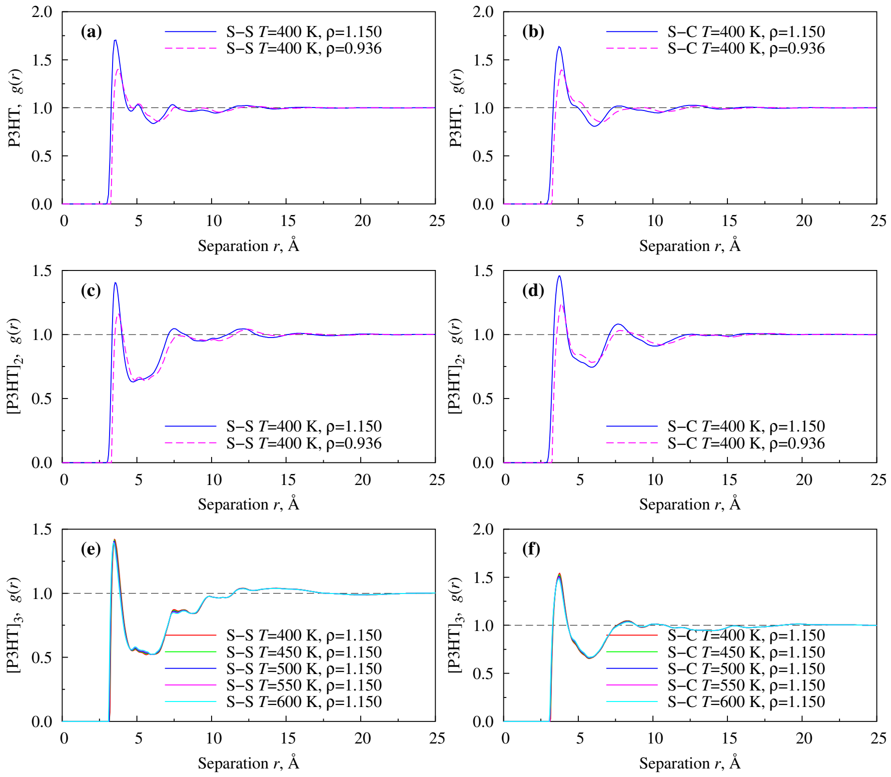

Figure S3. Selected RDFs of interaction sites belonging to P3HT monomers, (**a**,**b**), dimers, (**c**,**d**); and trimers, (**e**,**f**). Here S is the ring sulfur and C is the tail end carbon of the P3HT repeat unit (Figure S2a, sites #3 and #22, respectively). The effect of the density is well observed for monomers and dimers. For trimers, the physically meaningful solutions to RISM equations were found for only the higher density. The effect of the temperature for RDFs is rather insignificant, but for completeness it is included and demonstrated for P3HT trimers.

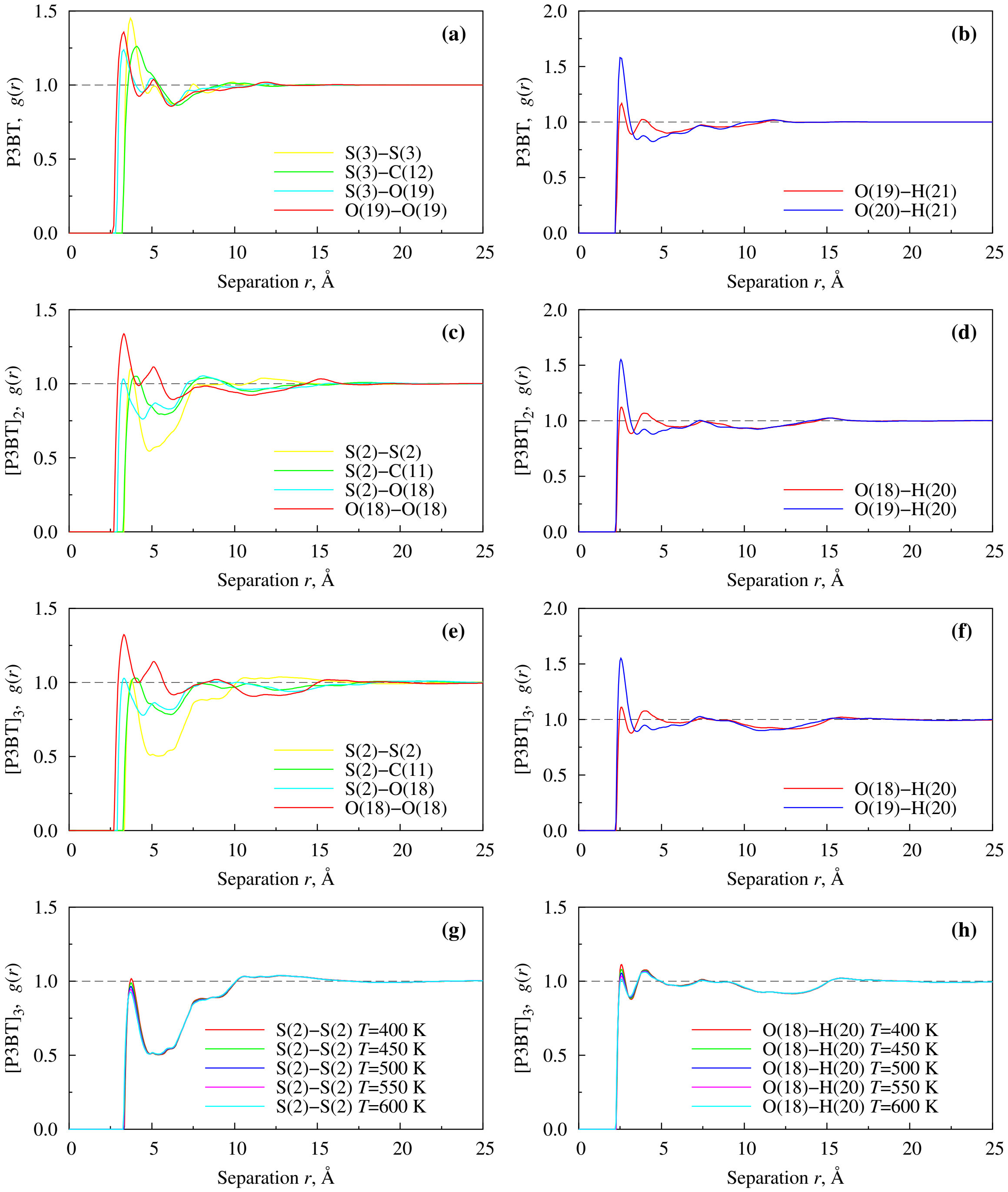

Figure S4. Selected RDFs of interaction sites belonging to P3BT monomers, (**a**,**b**), dimers, (**c**,**d**); and trimers, (**e**–**h**). Interaction sites appear with their number, which makes easier to identify them with the help of Figure S2b. The effect of the density is not seen as the physically meaningful solutions to RISM equations were found for only the higher density. Similarly to the P3HT system, the effect of the temperature for RDFs is rather insignificant, but for completeness it is included and demonstrated for P3BT trimers.

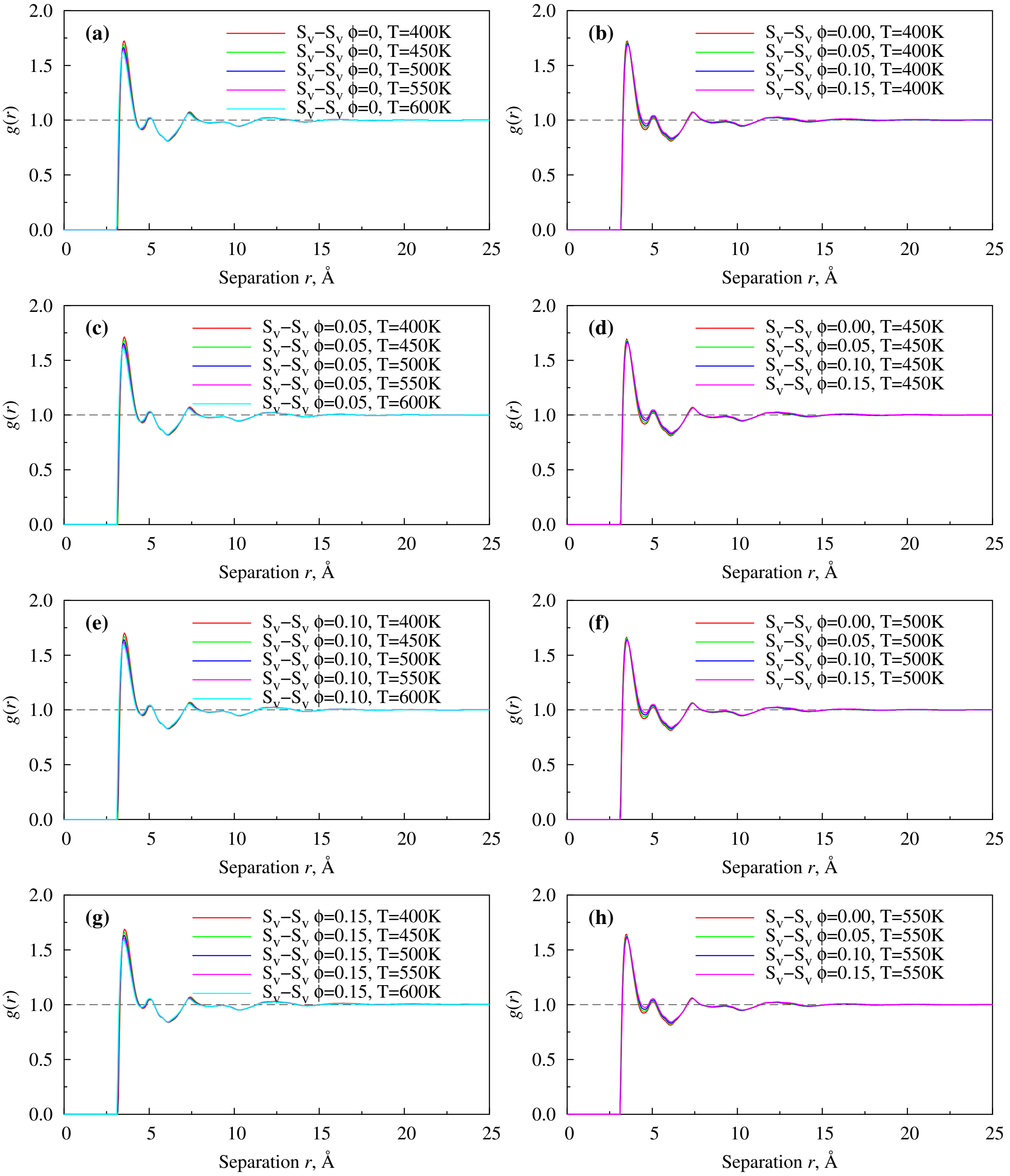

Figure S5. Selected RDFs of interaction sites belonging to P3HT monomer (solvent) in its blend with PCBM (solute), calculated at different temperatures T and solute weight fractions ϕ. S is the ring sulfur of the P3HT repeat unit (Figure S2a, site #3).

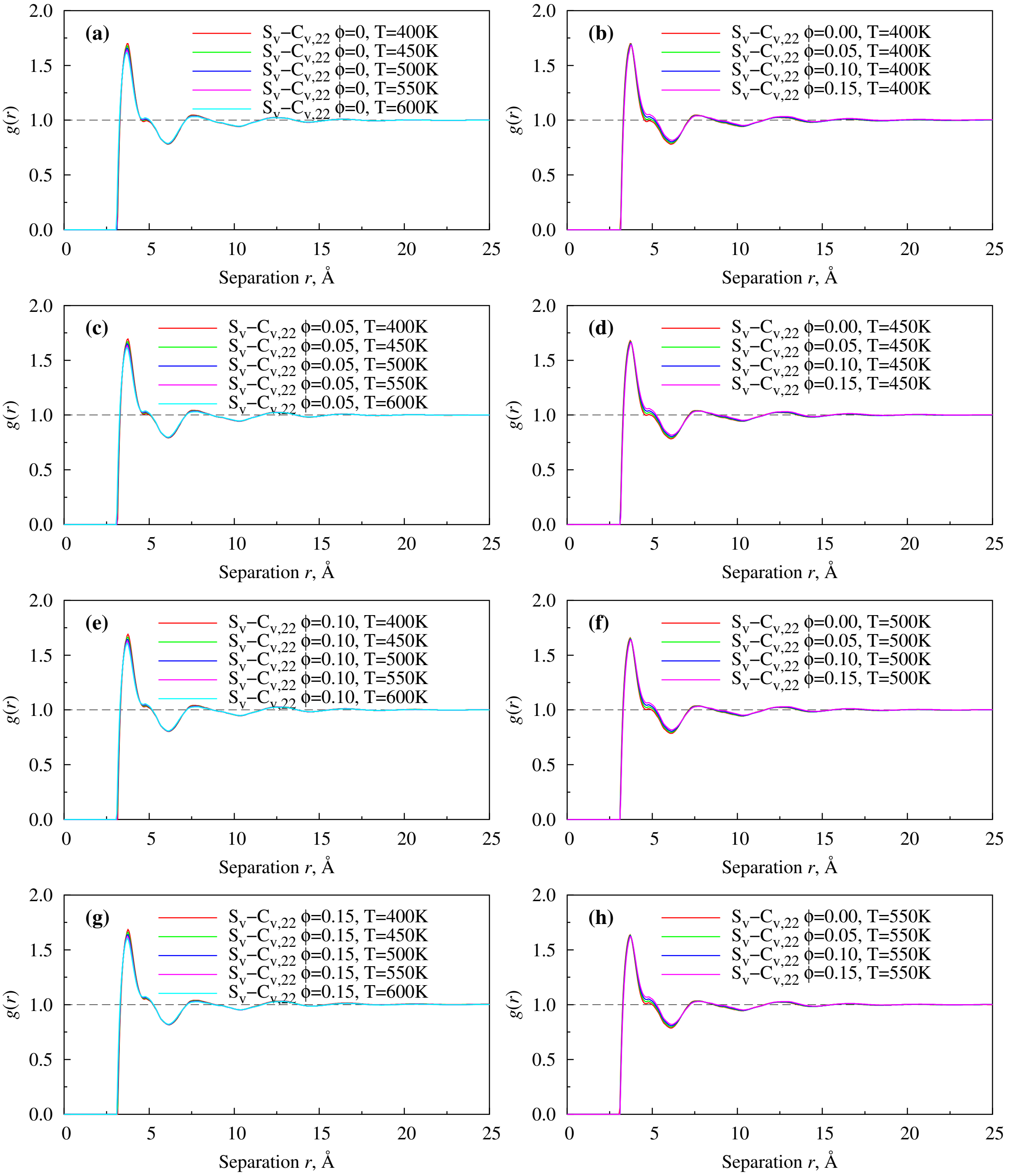

Figure S6. Selected RDFs of interaction sites belonging to P3HT monomer (solvent) in its blend with PCBM (solute), calculated at different temperatures T and solute weight fractions ϕ. Here S is the ring sulfur and C is the tail end carbon of the P3HT repeat unit (Figure S2a, sites #3 and #22, respectively).

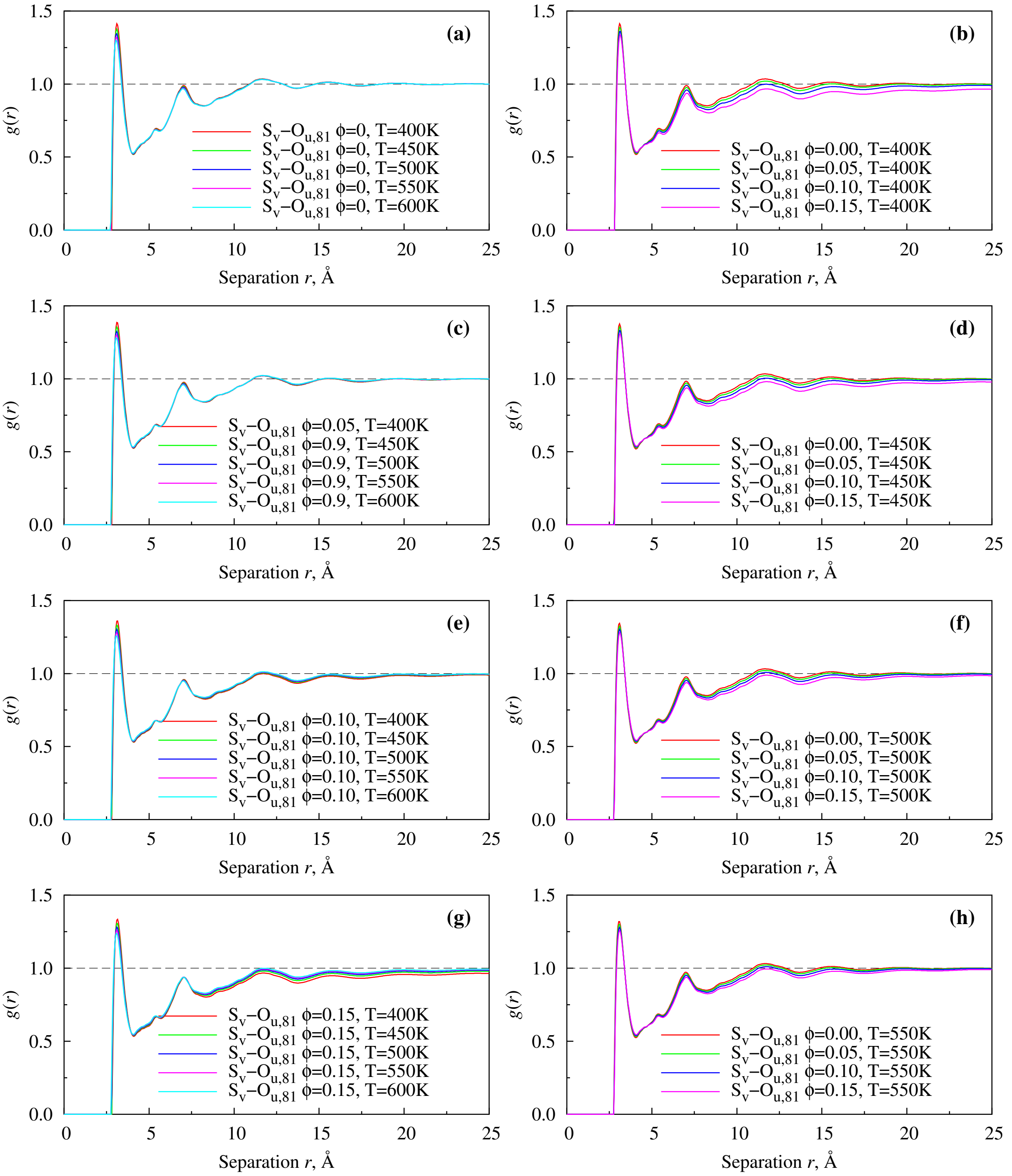

Figure S7. Selected RDFs of interaction sites belonging to P3HT monomer (solvent) and PCBM (solute), calculated at different temperatures T and solute weight fractions ϕ. Solute site and its id number are indicated for each plot.

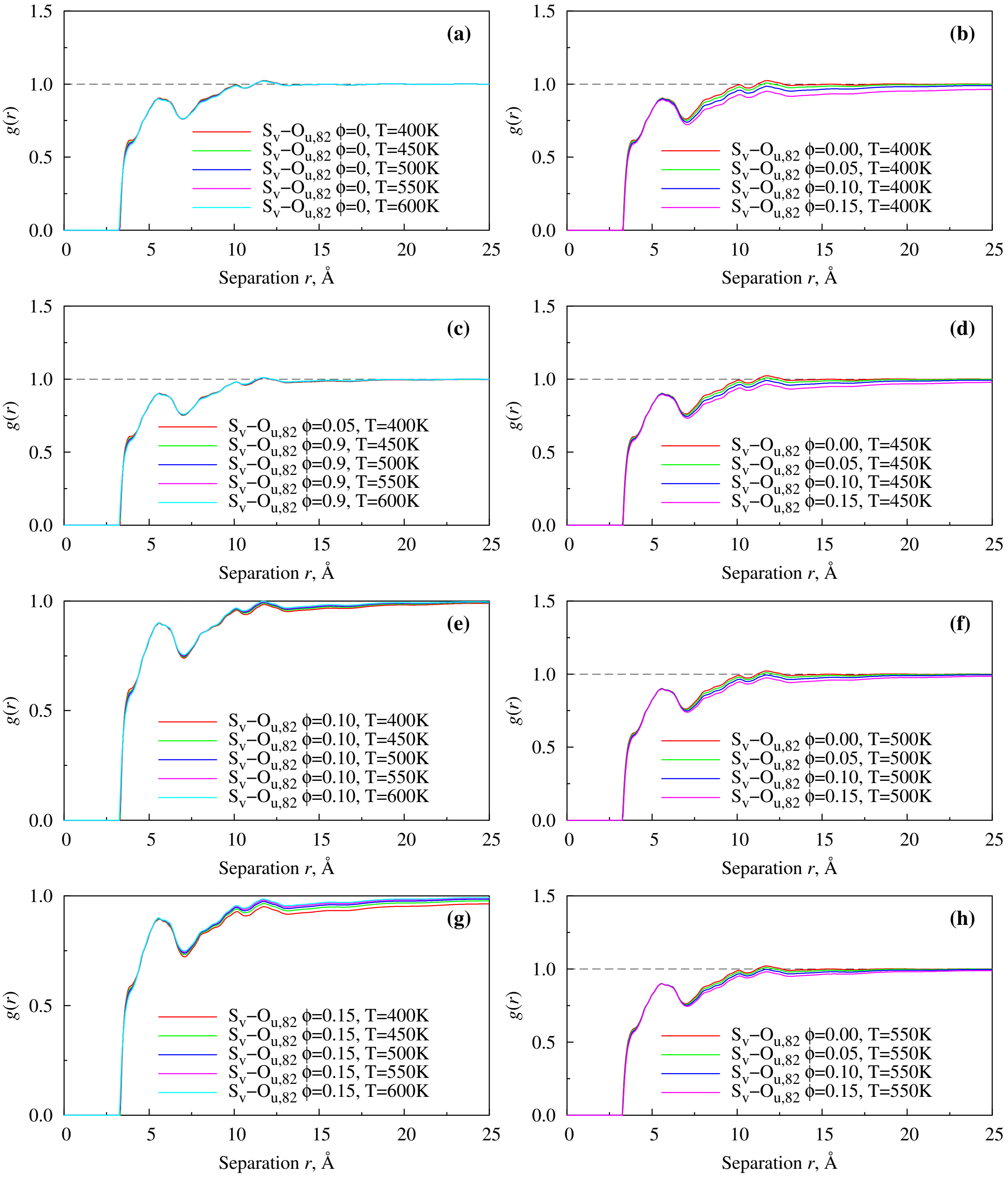

Figure S8. Selected RDFs of interaction sites belonging to P3HT monomer (solvent) and PCBM (solute), calculated at different temperatures T and solute weight fractions ϕ. Solute site and its id number are indicated for each plot.

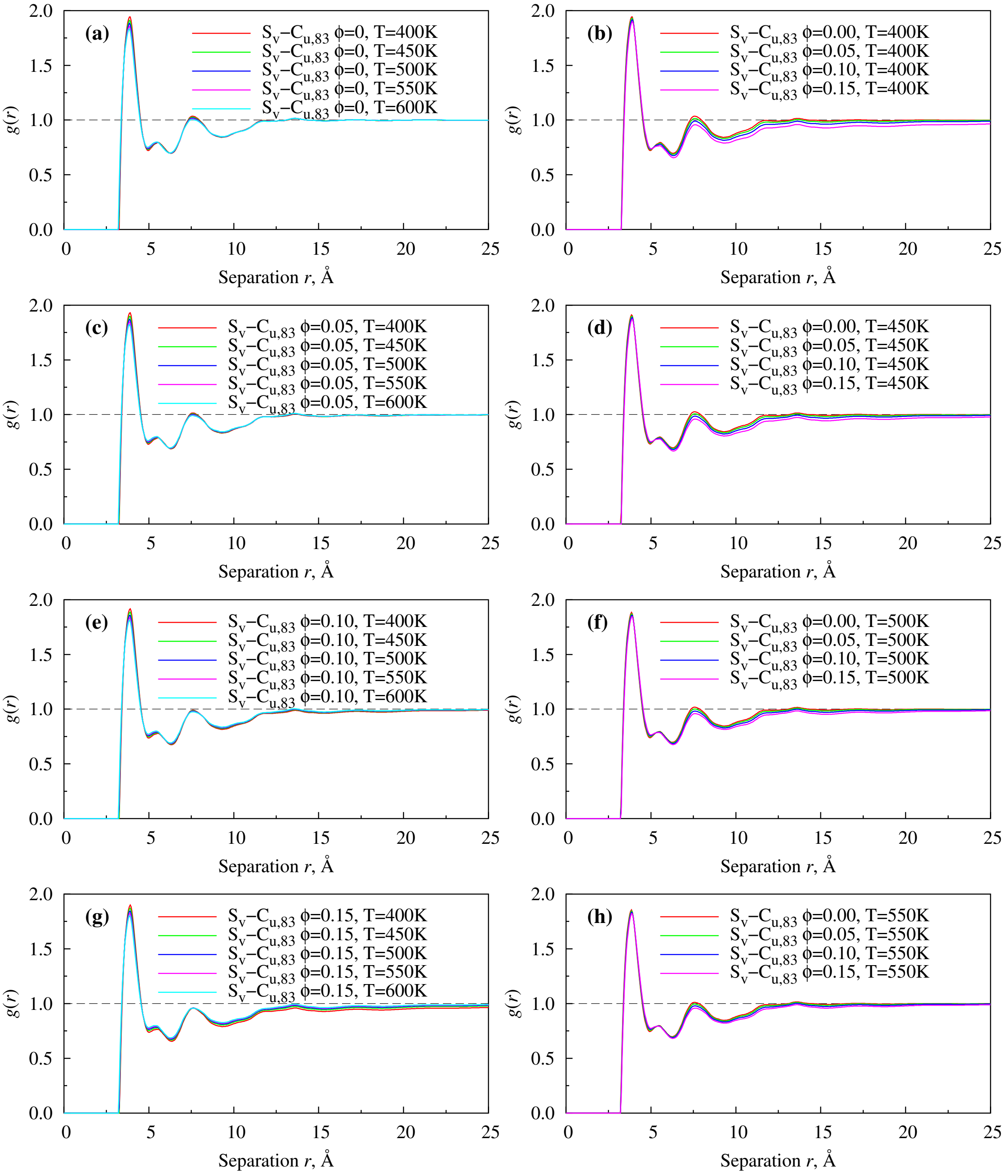

Figure S9. Selected RDFs of interaction sites belonging to P3HT monomer (solvent) and PCBM (solute), calculated at different temperatures T and solute weight fractions ϕ. Solute site and its id number are indicated for each plot.

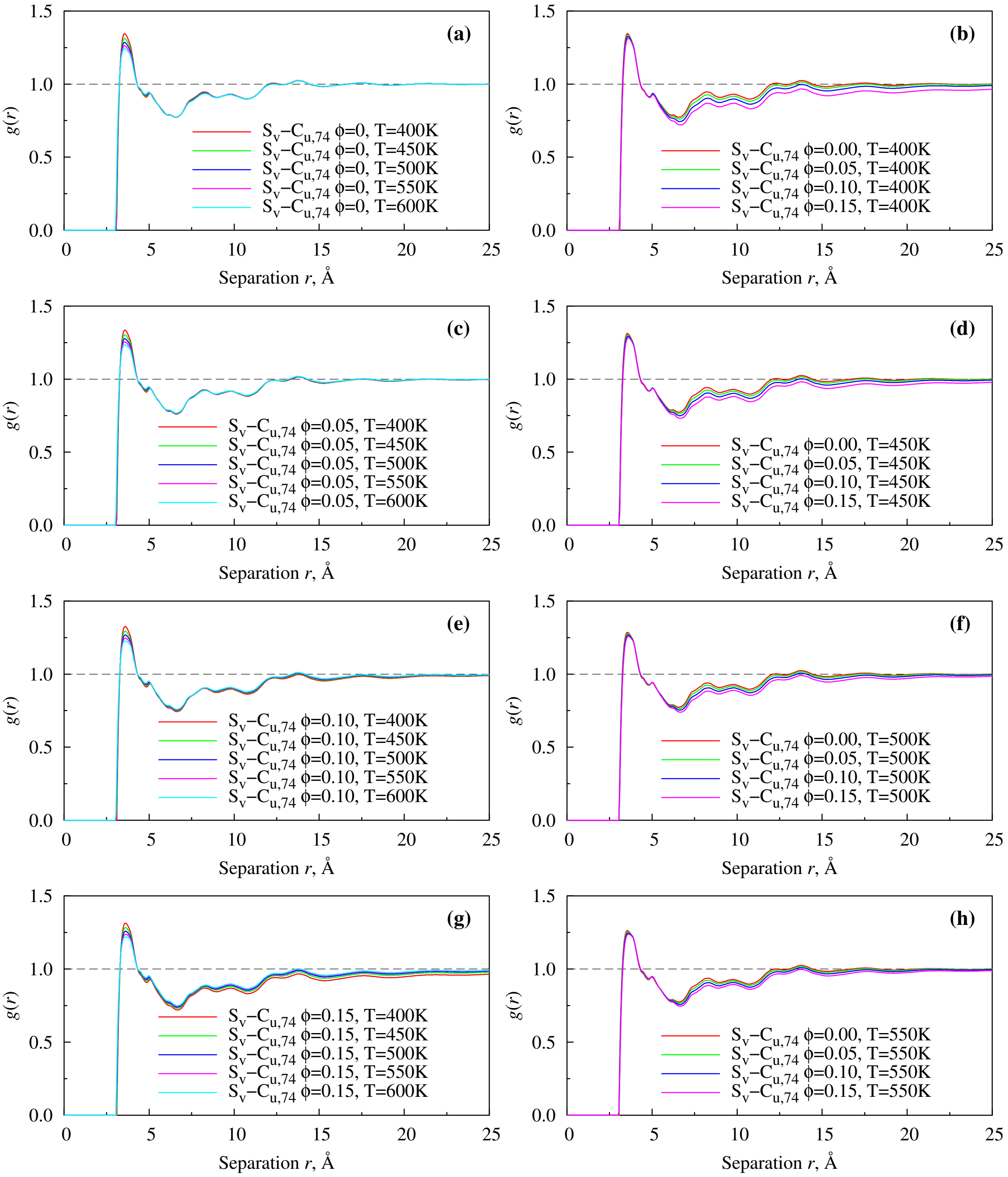

Figure S10. Selected RDFs of interaction sites belonging to P3HT monomer (solvent) and PCBM (solute), calculated at different temperatures T and solute weight fractions ϕ. Solute site and its id number are indicated for each plot.

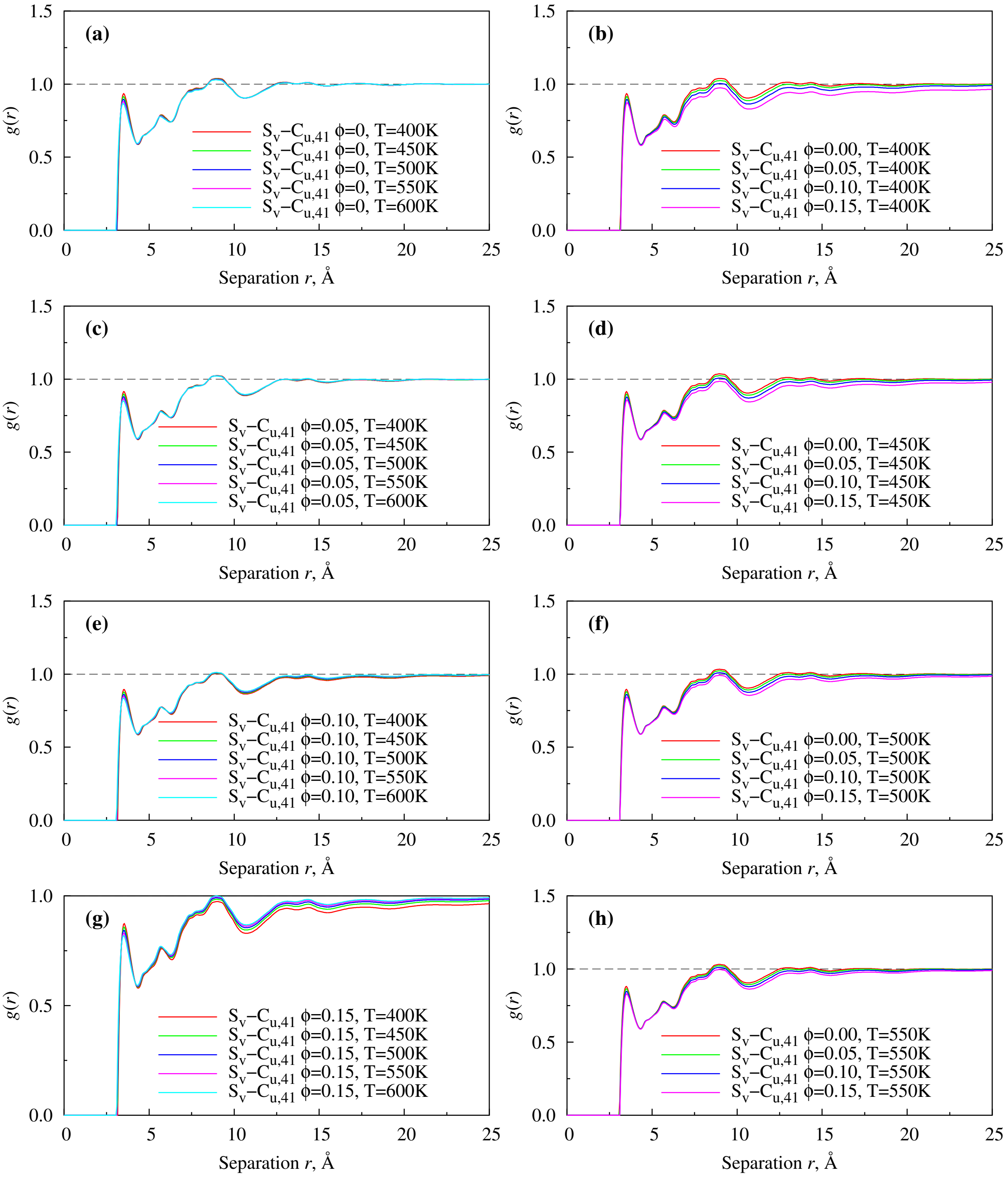

Figure S11. Selected RDFs of interaction sites belonging to P3HT monomer (solvent) and PCBM (solute), calculated at different temperatures T and solute weight fractions ϕ. Solute site and its id number are indicated for each plot.

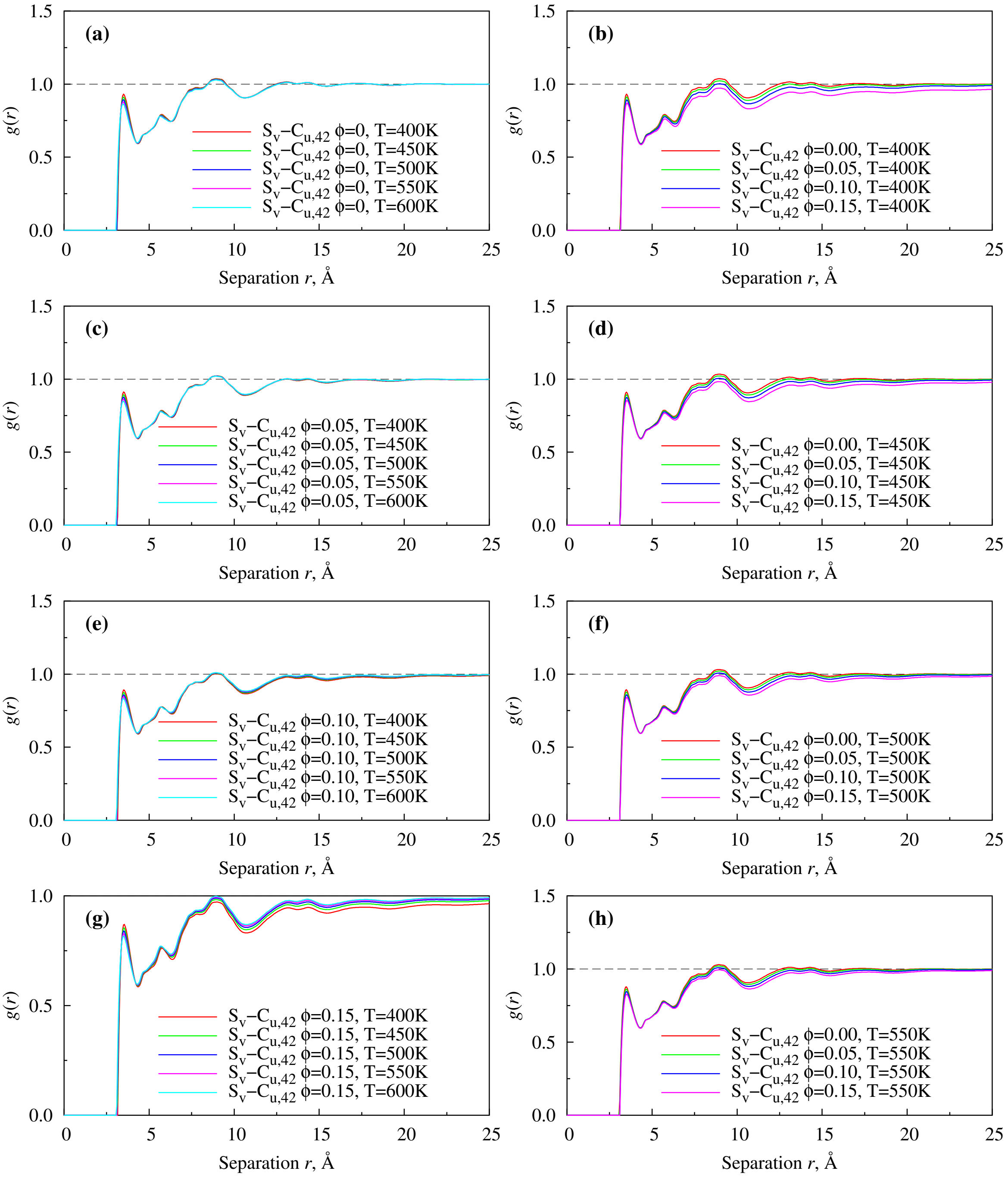

Figure S12. Selected RDFs of interaction sites belonging to P3HT monomer (solvent) and PCBM (solute), calculated at different temperatures T and solute weight fractions ϕ. Solute site and its id number are indicated for each plot.

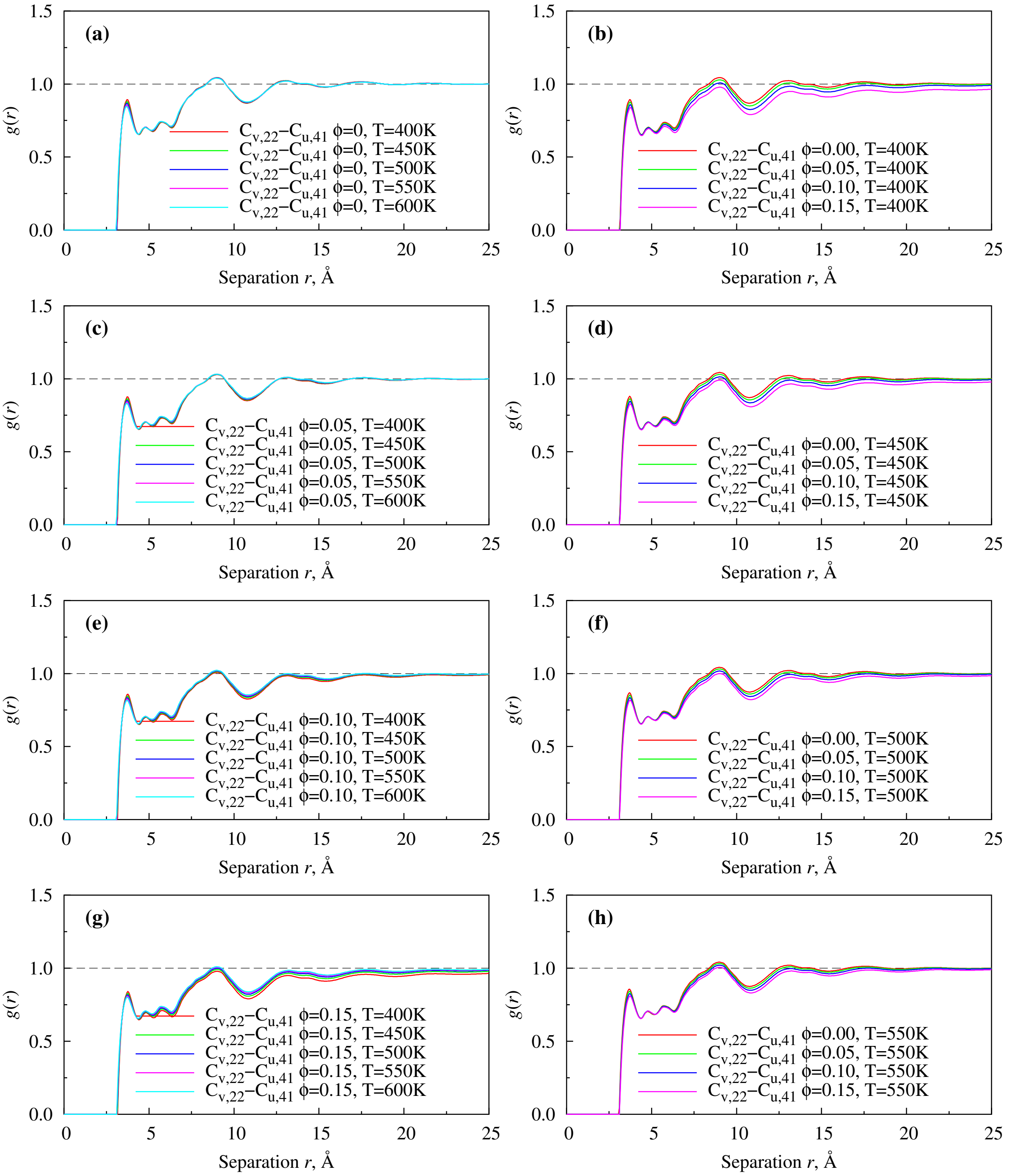

Figure S13. Selected RDFs of interaction sites belonging to P3HT monomer (solvent) and PCBM (solute), calculated at different temperatures T and solute weight fractions ϕ. Interaction sites and their id numbers are indicated for each plot.

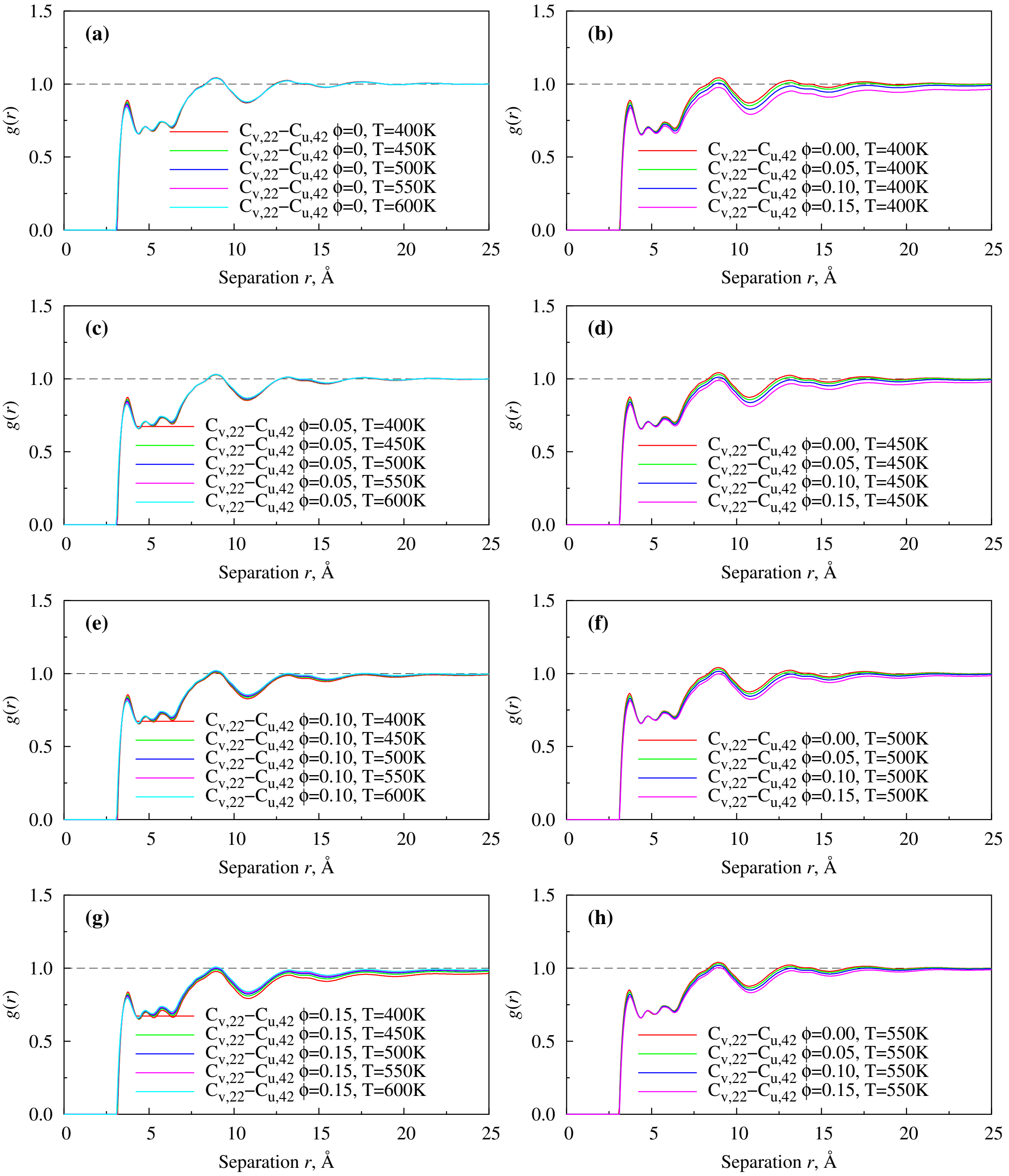

Figure S14. Selected RDFs of interaction sites belonging to P3HT monomer (solvent) and PCBM (solute), calculated at different temperatures T and solute weight fractions ϕ. Interaction sites and their id numbers are indicated for each plot.

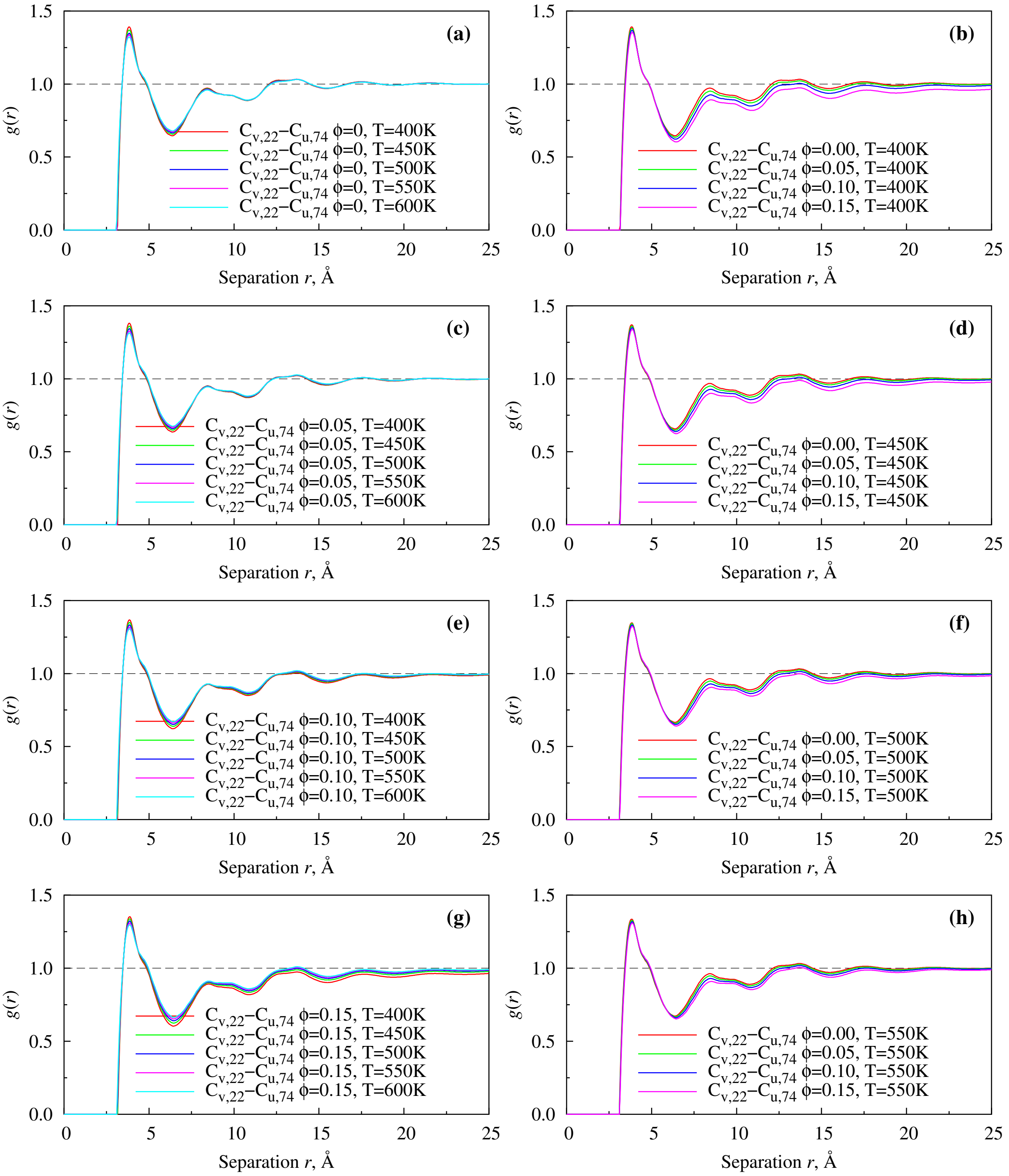

Figure S15. Selected RDFs of interaction sites belonging to P3HT monomer (solvent) and PCBM (solute), calculated at different temperatures T and solute weight fractions ϕ. Interaction sites and their id numbers are indicated for each plot.

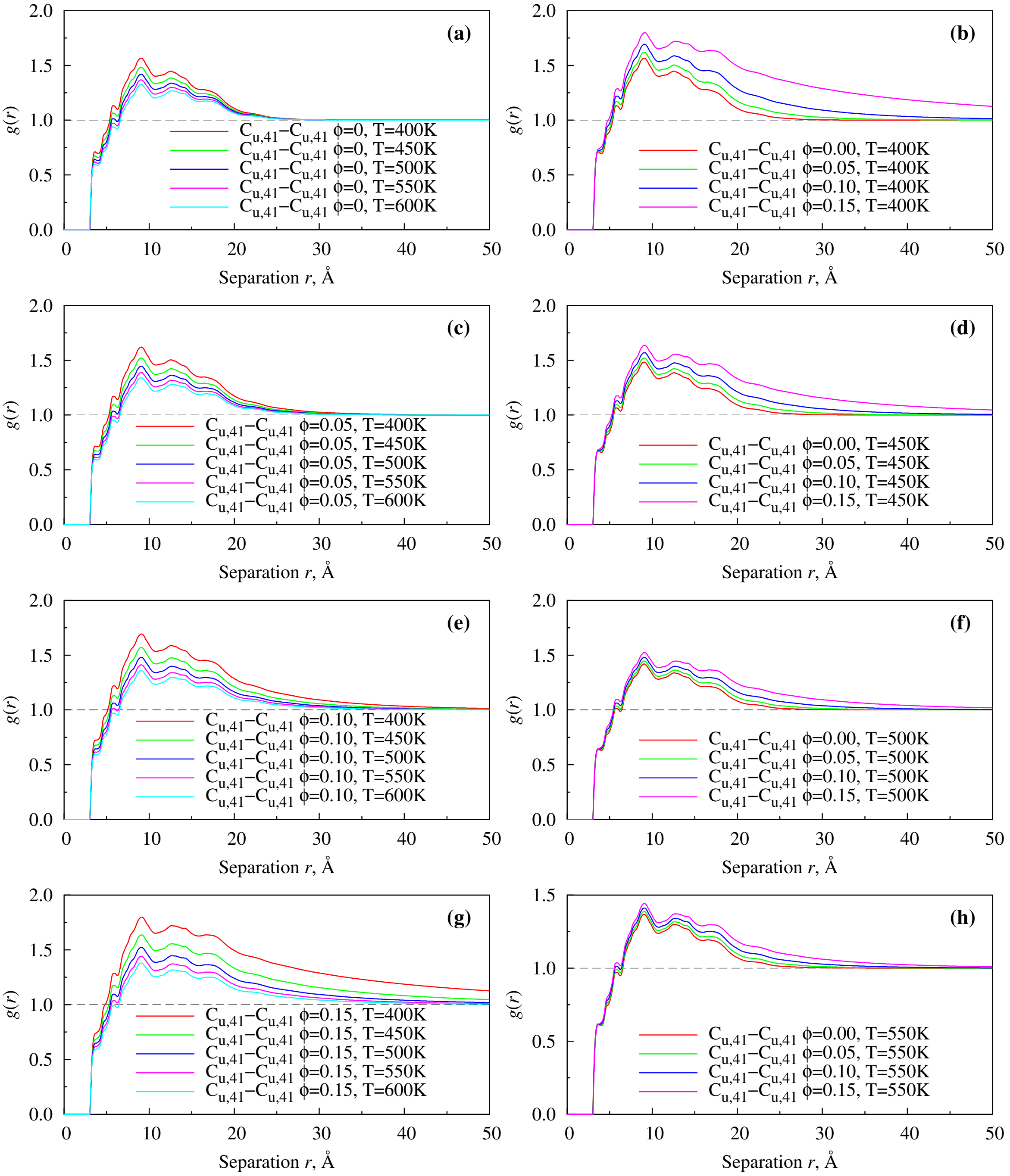

Figure S16. Selected RDFs of interaction sites belonging to PCBM (solute) in its blend with P3HT monomer (solvent), calculated at different temperatures T and solute weight fractions ϕ. Solute sites and their id numbers are indicated for each plot.

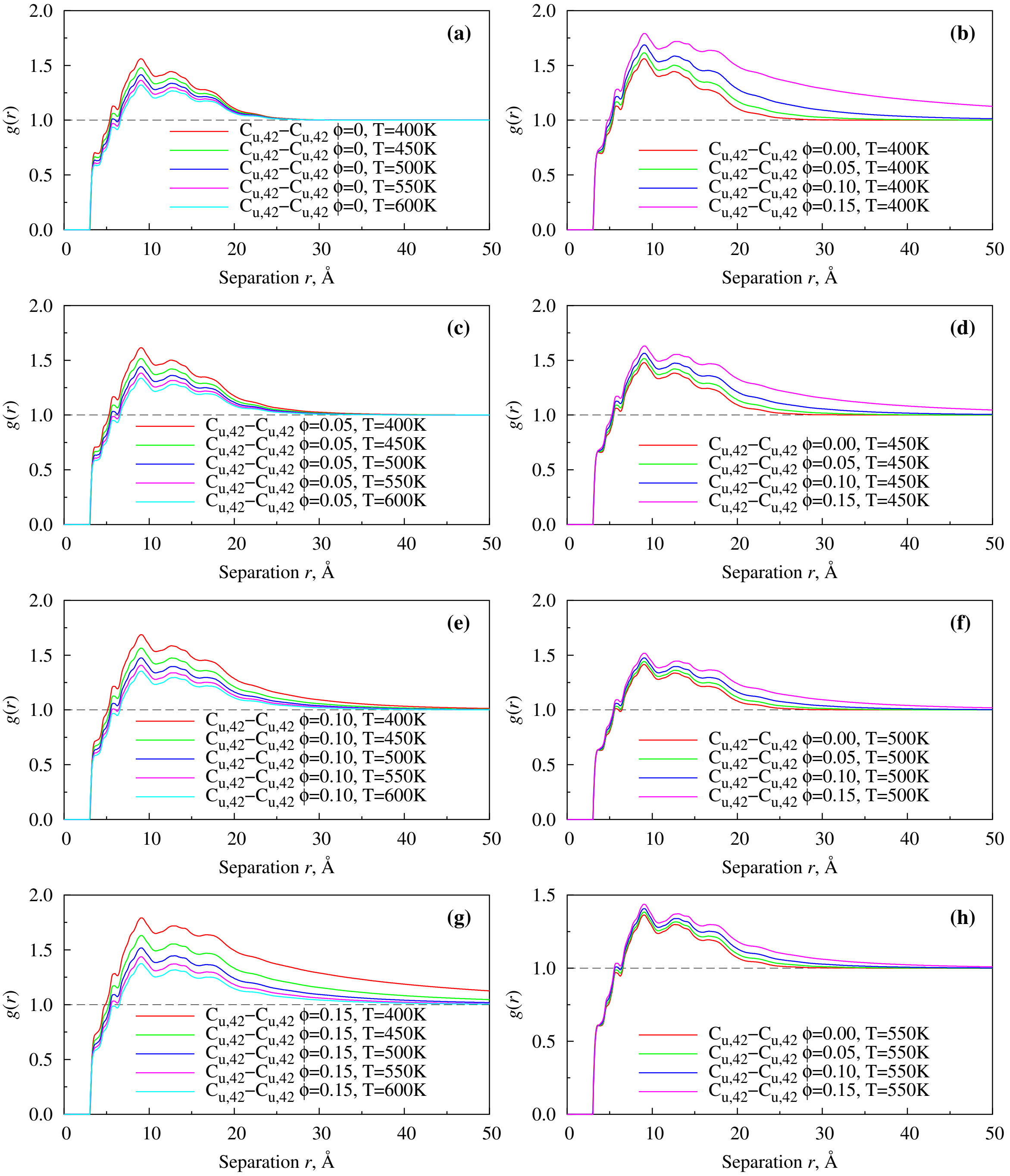

Figure S17. Selected RDFs of interaction sites belonging to PCBM (solute) in its blend with P3HT monomer (solvent), calculated at different temperatures T and solute weight fractions ϕ. Solute sites and their id numbers are indicated for each plot.

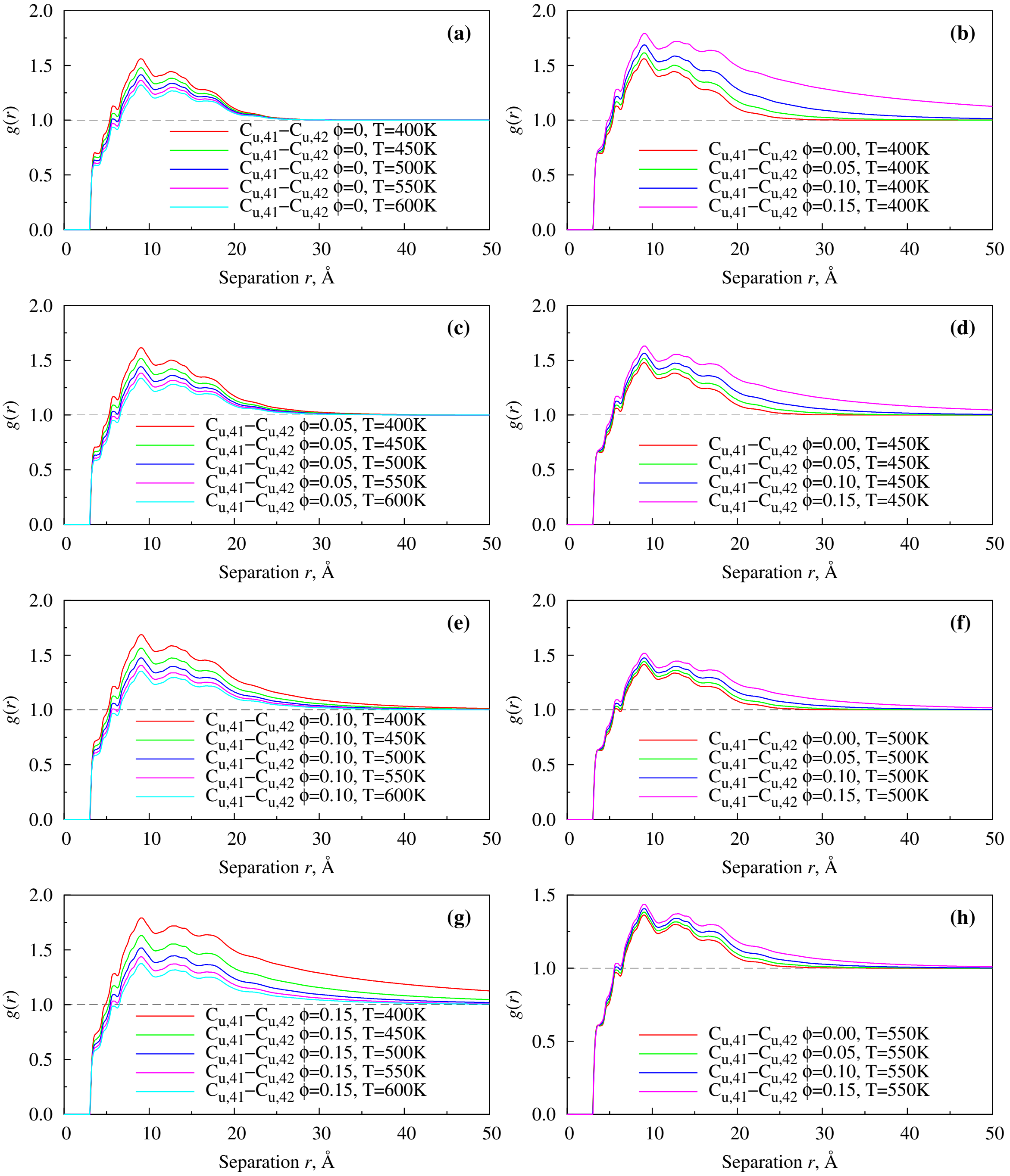

Figure S18. Selected RDFs of interaction sites belonging to PCBM (solute) in its blend with P3HT monomer (solvent), calculated at different temperatures T and solute weight fractions ϕ. Solute sites and their id numbers are indicated for each plot.

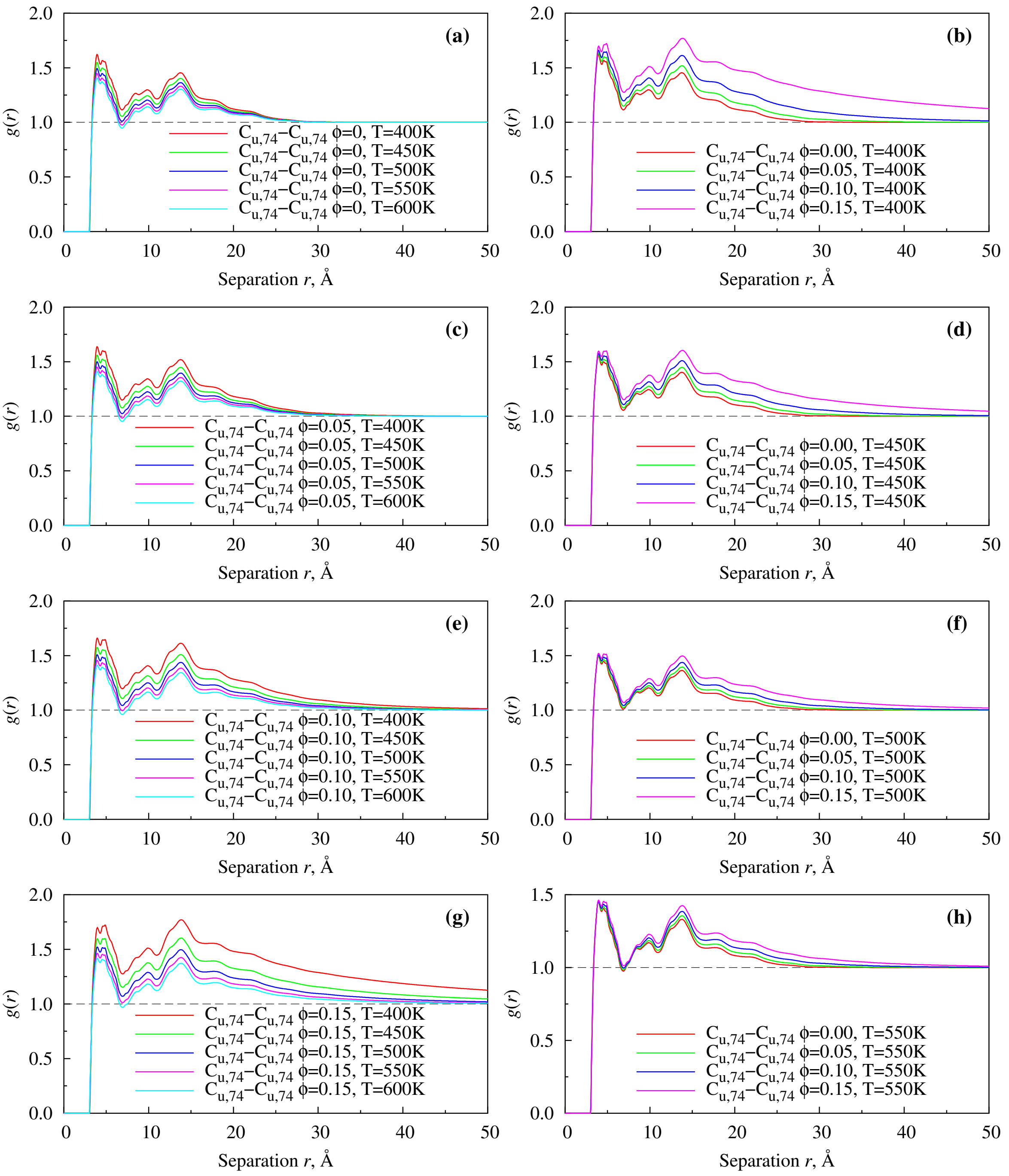

Figure S19. Selected RDFs of interaction sites belonging to PCBM (solute) in its blend with P3HT monomer (solvent), calculated at different temperatures T and solute weight fractions ϕ. Solute sites and their id numbers are indicated for each plot.

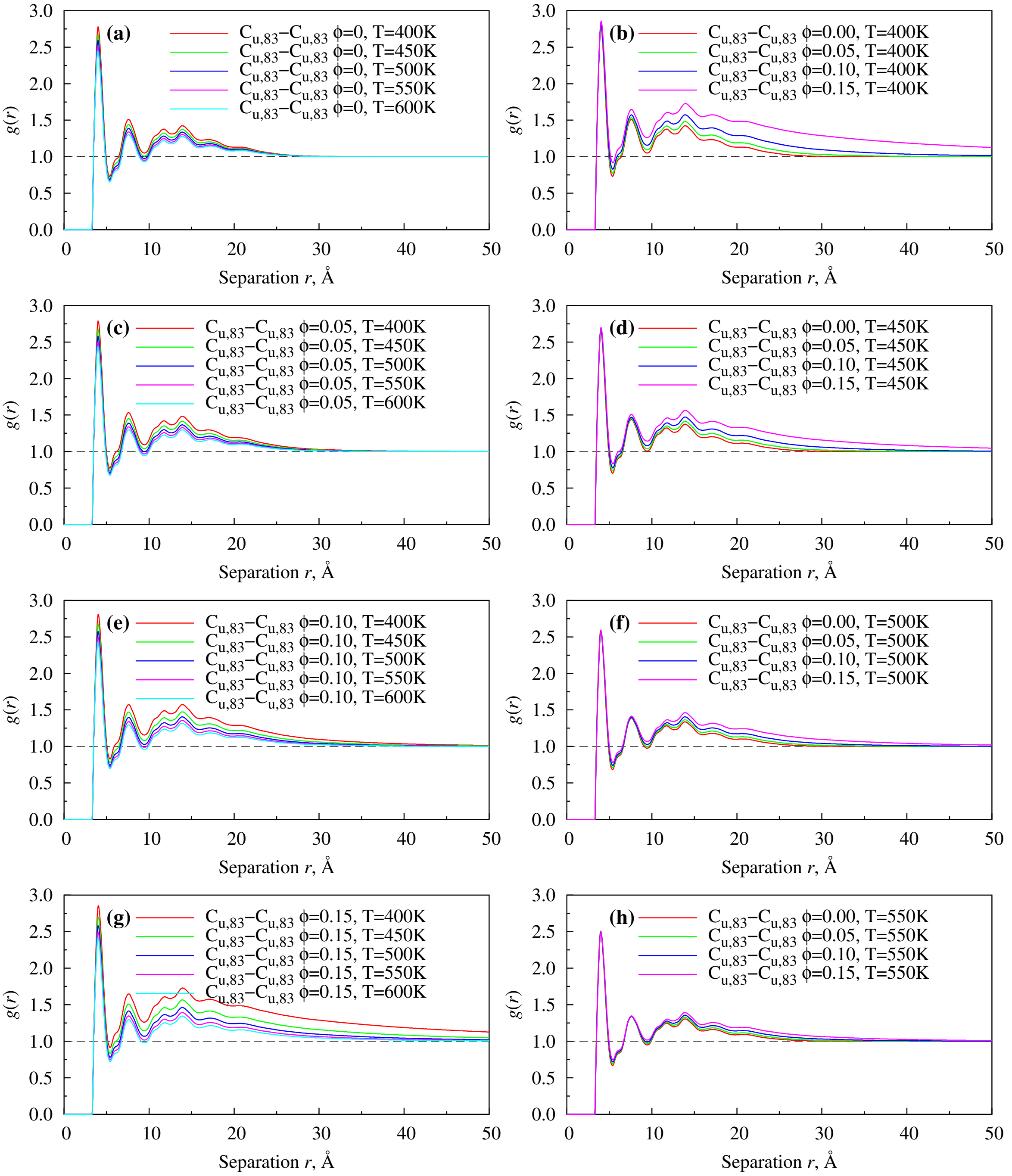

Figure S20. Selected RDFs of interaction sites belonging to PCBM (solute) in its blend with P3HT monomer (solvent), calculated at different temperatures T and solute weight fractions ϕ. Solute sites and their id numbers are indicated for each plot.

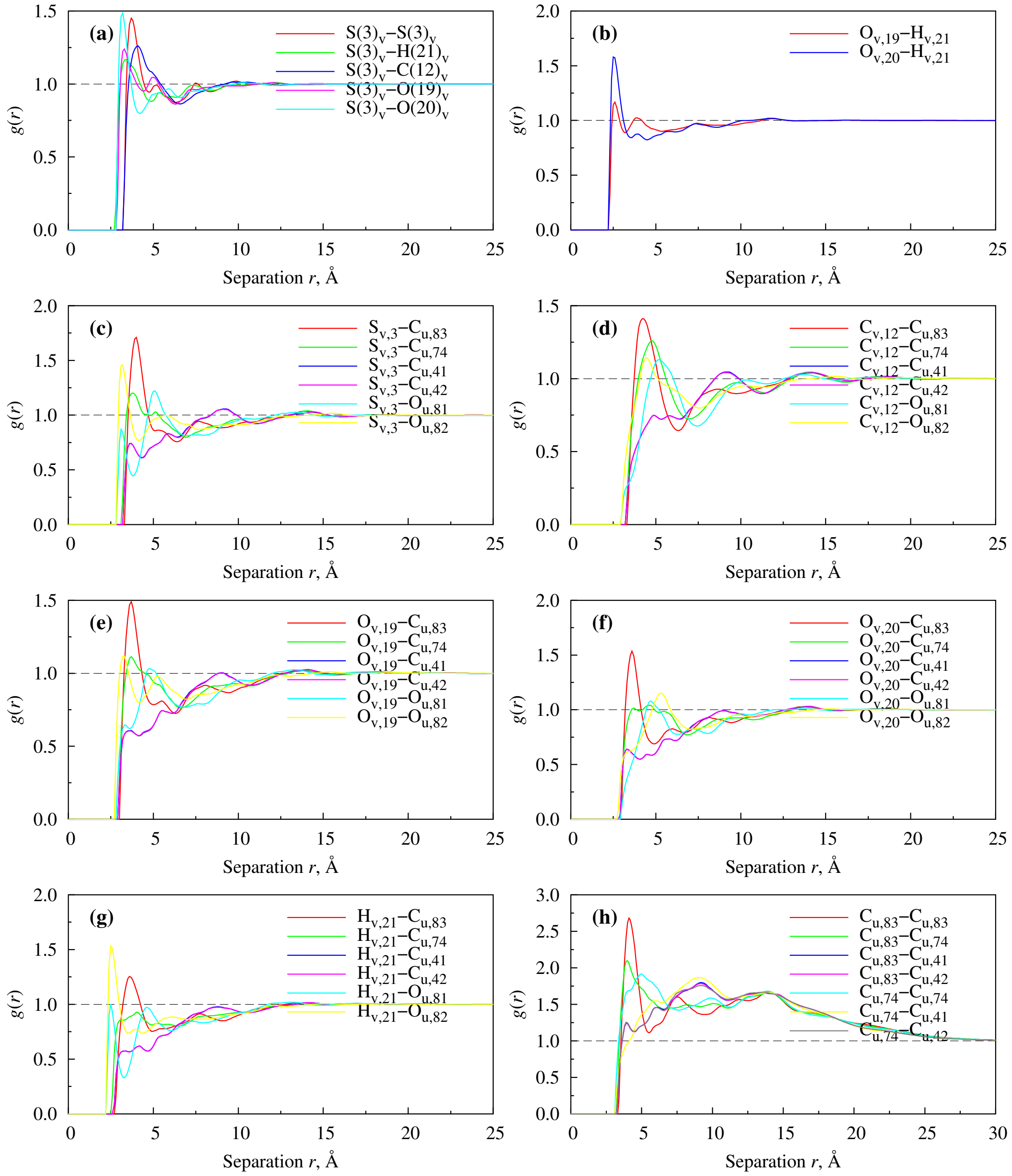

Figure S21. Selected RDFs of interaction sites belonging to P3BT monomer (solvent) and PCBM (solute) at infinite dilution, calculated at the temperature $T = 400$ K. All sites and their id numbers are indicated for each plot.

References

1. Rappe, A.K.; Casewit, C.J.; Colwell, K.S.; Goddard, W.A.; Skiff, W.M. Dissipative Particle Dynamics with an Effective Pair Potential from Integral Equation Theory of Molecular Liquids. *J. Am. Chem. Soc.* **1992**, *114*, 10024–10035.
2. Casewit, C.J.; Colwell, K.S.; Rappe, A.K. Application of a universal force field to organic molecules. *J. Am. Chem. Soc.* **1992**, *114*, 10035–10046.
3. Casewit, C.J.; Colwell, K.S.; Rappe, A.K. Application of a universal force field to main group compounds. *J. Am. Chem. Soc.* **1992**, *114*, 10046–10053.
4. Kobryn, A.E.; Nikolić, D.; Lyubimova, O.; Gusarov, S.; Kovalenko, A. Dissipative Particle Dynamics with an Effective Pair Potential from Integral Equation Theory of Molecular Liquids. *J. Phys. Chem. B* **2014**, *118*, 12034–12049.
